# Unequal burdens: a scoping review of key social determinants of health affecting wellbeing of international vs. domestic students

**DOI:** 10.1186/s12889-025-26152-z

**Published:** 2026-01-16

**Authors:** Nadia Farnaz, Taylah Scutts, Gantsetseg Ganbold, Putu Novi Arfirsta Dharmayani, Rimante Ronto, Seema Mihrshahi

**Affiliations:** 1 Department of Health Sciences, Faculty of Medicine, Health and Human Sciences, Macquarie University, Wallumattagal Campus Macquarie Park, Level 5, 75 Talavera Road, North Ryde, Sydney, NSW 2109 Australia; 2 School of Public Health, The University of Sydney, Sydney, 2006 Australia

**Keywords:** Social determinants of health, Food insecurity, Housing, Social isolation, Psychological distress, Wellbeing, International students, Domestic students, Higher education

## Abstract

**Background:**

The wellbeing of university students has become a significant concern globally. This scoping review aimed to explore key social determinants of health, such as food insecurity, housing issues, social isolation, and psychological distress and how it affects the overall experiences and wellbeing of university students. The review included studies comparing the experiences of international and domestic students to highlight unique challenges faced by each group.

**Methods:**

This scoping review followed the first five stages of Arksey and O’Malley’s framework and used Preferred Reporting Items for Systematic Reviews and Meta-Analyses extension for Scoping Reviews (PRISMA-ScR) checklist. A comprehensive search was conducted across Medline, Scopus, and Web of Science databases, focusing on studies published between 2014 and 2024. The inclusion criteria targeted research on international students in comparison to domestic students addressing food insecurity, housing issues, social support, psychological distress and wellbeing. A total of 23 studies met the inclusion criteria and were assessed for quality using the Joanna Briggs Institute (JBI) critical appraisal tool. Data were extracted and synthesised using a narrative approach.

**Results:**

The review identified that international students are particularly vulnerable to psychological distress due to combined influence of other key social determinants of health. Social isolation was identified as a critical factor, with international students reporting higher levels of loneliness and lack of social support compared to domestic counterparts. Food insecurity significantly impacts both domestic and international students, with international students experiencing higher levels of anxiety and depression due to limited access to affordable food and support networks. Housing issue affect stress and mental health, particularly among international students who face additional barriers such as cultural adjustments and unfamiliarity with local housing markets. The interconnectedness of these factors intensifies their impact, resulting in poorer wellbeing among international students compared to domestic students.

**Conclusions:**

This review highlights the critical need for targeted interventions to address food insecurity, housing issues, social isolation and psychological distress among university students, particularly international students. Comprehensive support systems and tailored policies are recommended, and future research is needed to develop and evaluate solutions for these challenges to improve student wellbeing.

**Supplementary Information:**

The online version contains supplementary material available at 10.1186/s12889-025-26152-z.

## Background

The wellbeing of university students globally is a significant concern, with growing prevalence of psychological distress [1, 2]. Wellbeing, defined by interconnection of good health, life satisfaction and positive mental, social and physical health, is inversely related to psychological distress, which involves emotional suffering and discomfort manifesting anxiety and depression [3–6]. Social Determinants of Health (SDoH) such as access to food, housing, education, employment, healthcare, and social support, are crucial in shaping an individual’s wellbeing by either strengthening or undermining it [5, 7, 8].

University students, especially international, face heightened challenges like food insecurity, housing issues, social isolation, and psychological distress [9, 10]. Here, food insecurity refers to a lack of consistent access to nutritious food [11]; housing issues involve a shortage of secure and affordable housing [12]; and social isolation is characterised by limited social interaction and feeling of loneliness [13–15]. These challenges may be intensified by their limited social support networks, language barriers, and unfamiliarity with local resources, contribute to a greater negative impact on the wellbeing of international students compared to domestic students [9, 10, 16, 17]. Understanding their psychological experiences and wellbeing in comparison to their domestic counterparts has become an area of considerable interest [7, 8, 18].

Research shows nearly 39% of university students worldwide experience symptoms of psychological distress, with a recent study indicating rising stress (84%), anxiety (36%) and depression (55%) among students which can impair their academic performance leading to lower self-worth, addiction, dropouts and worsened mental and physical health [9, 19–22]. International students report higher rates of distress (67%) compared to domestic students (45%) due to unique stressors like accommodation, managing finances, and balancing work, study, and personal life while adapting to tertiary education in a new country [16, 23, 24]. They are less likely to seek support due to stigma, language and cultural barriers, lack of knowledge, and self-restrictive attitudes [24]. Food insecurity and social isolation are particularly severe among international students, with studies revealing 74.4% of them face food insecurity compared to 22.7% of domestic students in Australia [23, 25, 26]. These challenges, along with housing shortages (affecting 69% of international students), and increased psychological distress, especially since the COVID-19 pandemic, have significantly impacted students’ overall wellbeing where younger students and females have been particularly affected [23, 27–31].

While the existing research provides valuable insights into the challenges faced by international students, there are still several aspects of this topic that remain unknown. Food insecurity, anxiety, depression, and stress are closely linked, while housing issues further contribute to stress and negatively impacts academic performance [21, 23, 25, 27]. Additionally, the lack of robust social support networks intensifies feelings of loneliness and isolation [6]. These factors are often more pronounced for international students who face additional challenges in navigating local resources and support systems compared to their domestic counterparts. The interconnection of these social determinants of health might amplify their overall impact on student’s wellbeing. Additionally, the specific intersectionality of cultural adjustment, language barriers, and discrimination, as experienced by international students from diverse backgrounds, is an important aspect that warrants deeper investigation [32]. A comprehensive understanding of the complex influence of these social determinants on one another and overall wellbeing is essential for designing and developing effective and appropriate interventions [33].

In order to gain a thorough understanding of the existing literature, this scoping review aimed to understand the complex interplay within the key social determinants of health and their impact on university students depending on student status (international vs. domestic). In particular, the review examined how these factors influence international students’ experiences and wellbeing in comparison to domestic students. The following research question guided this scoping review: How do the key social determinants of health (food insecurity, social isolation, housing issues, psychological distress) affect the overall experiences and wellbeing of international versus domestic students?

## Methods

A scoping review was selected as the most appropriate method as it provided the opportunity to explore and synthesise a more comprehensive evidence base of literature. It systematically maps the literature on key social determinants of health affecting international and domestic students’ wellbeing. Unlike an integrative review, which synthesises findings to draw conclusions, a scoping review identifies key themes, diverse methodologies, and knowledge gaps [34]. Given the broad research scope and interdisciplinary nature of the topic, this approach allows for the inclusion of varied study designs, ensuring a comprehensive understanding. Additionally, as this is an emerging research area with fragmented evidence, a scoping review helps identify knowledge gaps and establish a foundation for future systematic reviews or meta-analyses.

The review methods were guided by the six-stage framework for scoping reviews developed by Arksey and O’Malley [35]. This review adopted the first five stages of this framework, encompassing the identification of the research question, relevant studies, selection of studies, data charting, and collation, summarisation, and reporting of results. The scoping review adhered to the criteria outlined by the Preferred Reporting Items for Systematic Reviews and Meta-Analyses extension for Scoping Reviews (PRISMA-ScR) guideline [36]. Comprehensive details of the study methodology and search strategy are outlined in the scoping review protocol, which is registered with the Open Science Framework (https://osf.io/8mvh5/). The inclusion criteria in the protocol initially specified studies from 2015 to 2024, reflecting this review’s aim to focus on recent research. However, the search was expanded to include studies from 2014 to ensure a more comprehensive review, although no studies from 2014 were ultimately included.

### Conceptual framework

**Fig. 1:**
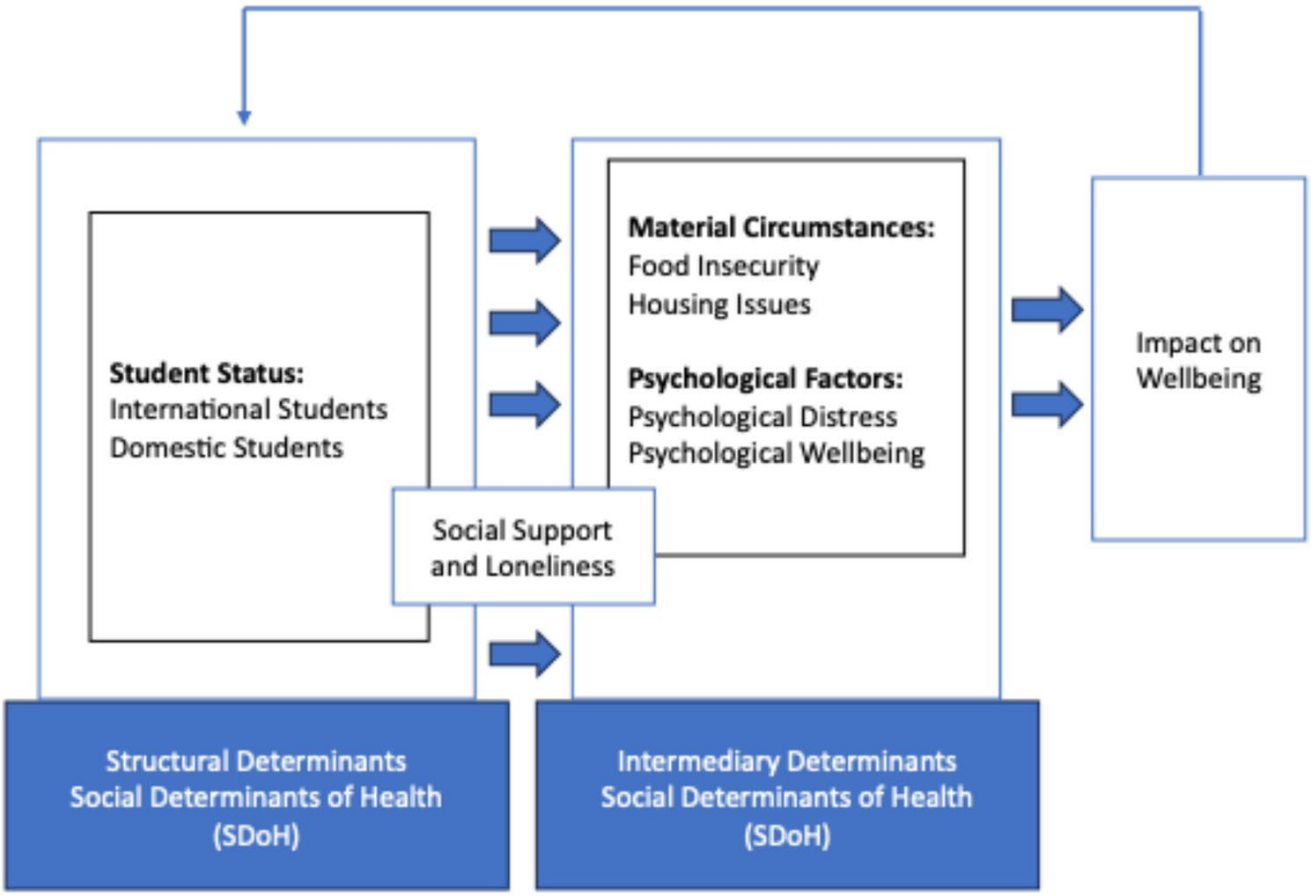
Adaptation of WHO Commission on the SDoH [37]

This review, following the WHO Commission on the Social Determinants of Health (CSDH) framework, analyses how key structural (student status) and intermediary SDoH (food insecurity, housing issues, psychological distress, wellbeing) along with social cohesion factors (social support, loneliness) collectively impact university students’ overall wellbeing, while other important SDoH (e.g., employment, income, academic stress) are beyond this study’s scope [37]. Figure 1 below provides an overview of this adopted framework.

### Operational definition

For this scoping review, wellbeing is operationally defined as positive experiences resulting from key SDoH, including psychological resilience, food security, stable housing, and social inclusion and support, which collectively contribute to comprehensive understanding of wellbeing. Additionally, the term ‘international students’ refers to individuals who have moved to a foreign country to pursue higher degree or tertiary education [38]. The term ‘domestic students’ refers to individuals who are native to the country in which they are studying and pursuing higher education [38]. All the published peer reviewed studies in this scoping review used the term international students but some of the studies referred to domestic students as local or native students.

### Search strategy

The research team, in consultation with an academic liaison librarian, developed the search strategy by deriving broad concepts and keywords from the research question. For this review, three databases were selected (Medline, Scopus, and Web of Science) for their extensive health, social sciences, and multidisciplinary research coverage. Medline provides public health and biomedical studies, Scopus captures interdisciplinary and policy-related research, and Web of Science ensures high-quality, peer-reviewed sources. This combination maximises relevant study retrieval while minimising redundancy, ensuring a robust and comprehensive search strategy. The research team systematically searched through these three databases (Medline, Scopus, and Web of Science) and identified peer-reviewed studies from 1 January 2014 to 29 February 2024. Medical subject heading (MeSH) terms and keywords were selected accordingly. A sample search strategy is detailed in Table 1.

**Table 1:** Medline search strategy (05.06.2024)

No.	Searches	Results
1	Stress, psychological/	135,822
2	(psychological stress or psychological distress or emotional stress or emotional distress or mental anguish or depression or anxiety).mp. [MP = title, abstract, original title, name of substance word, subject heading word, floating sub-heading word, keyword heading word, organism supplementary concept word, protocol supplementary concept word, rare disease supplementary concept word, unique identifier, synonyms]	739,088
3	1 or 2	824,581
4	wellbeing/or mental health/or wellness/or happiness/or satisfaction/or health/or quality of life/	397,613
5	(wellbeing or mental health or wellness or happiness or satisfaction or health* or quality of life).mp. [MP = title, abstract, original title, name of substance word, subject heading word, floating sub-heading word, keyword heading word, organism supplementary concept word, protocol supplementary concept word, rare disease supplementary concept word, unique identifier, synonyms]	5,307,824
6	4 or5	5,307,824
7	3 and 6	328,264
8	food insecurity/or access to healthy foods/or food security/or hunger/or food supply/	23,042
9	(food adj2 (secur* or insecur* or poverty or hunger or scarcity or access or availab* or afford*)).mp. [MP = title, abstract, original title, name of substance word, subject heading word, floating sub-heading word, keyword heading word, organism supplementary concept word, protocol supplementary concept word, rare disease supplementary concept word, unique identifier, synonyms]	34,397
10	social isolation/or ostracism/or loneliness/or social exclusion/or social connect*/	22,169
11	(social isolation or social exclusion or ostracis* or loneliness or social connect*).mp. [MP = title, abstract, original title, name of substance word, subject heading word, floating sub-heading word, keyword heading word, organism supplementary concept word, protocol supplementary concept word, rare disease supplementary concept word, unique identifier, synonyms]	44,397
12	housing instability/or housing issue/or living condition/	30,227
13	(housing crisis or accomodation crisis or housing scarcity or residential crisis or living condition or housing).mp. [MP = title, abstract, original title, name of substance word, subject heading word, floating sub-heading word, keyword heading word, organism supplementary concept word, protocol supplementary concept word, rare disease supplementary concept word, unique identifier, synonyms]	64,172
14	“Social Determinants of Health”/	7365
15	social determinants of health.mp. [MP = title, abstract, original title, name of substance word, subject heading word, floating sub-heading word, keyword heading word, organism supplementary concept word, protocol supplementary concept word, rare disease supplementary concept word, unique identifier, synonyms]	17,694
16	8 or 9 or 10 or 11 or 12 or 13 or 14 or 15	169,949
17	Universities/	54,862
18	(universit* or college* or tertiary or higher education*).mp. [MP = title, abstract, original title, name of substance word, subject heading word, floating sub-heading word, keyword heading word, organism supplementary concept word, protocol supplementary concept word, rare disease supplementary concept word, unique identifier, synonyms]	984,801
19	17 or 18	984,801
20	Students/	85,313
21	(student* or university student* or college student* or undergraduate or postgraduate or graduate or senior).mp. [MP = title, abstract, original title, name of substance word, subject heading word, floating sub-heading word, keyword heading word, organism supplementary concept word, protocol supplementary concept word, rare disease supplementary concept word, unique identifier, synonyms]	540,746
22	(international student* or domestic student*).mp. [MP = title, abstract, original title, name of substance word, subject heading word, floating sub-heading word, keyword heading word, organism supplementary concept word, protocol supplementary concept word, rare disease supplementary concept word, unique identifier, synonyms]	1509
23	20 or 21 or 22	540,746
24	19 and 23	149,790
25	7 and 16 and 24	539
26	limit 25 to (english language and human and vr="2014-Current”)	329

### Eligibility criteria

This study’s scope included quantitative and qualitative primary empirical research studies published in a peer-reviewed journal between January 2014 and May 2024 and written in English. The study included studies published between 1 January 2014 and 29 February 2024 to ensure a comprehensive synthesis of recent literature. Although this review initially planned (during the protocol stage) to include studies published from 2015 onwards to focus on contemporary research reflecting recent global scenarios and their impact on key social determinants of health, the team decided to expand the timeline to include studies from 2014. This decision was made to ensure that no critical studies published just before the cutoff year of 2015 were missed. Ultimately, no studies from 2014 met the inclusion criteria, but the review retained this expanded timeline in the search to reflect the efforts to conduct an exhaustive review.

All studies included in this scoping review consisted of international and domestic students, as study population, enrolled in universities (tertiary education institutions). Only studies that included a comparative analysis between these two groups of students were considered. The primary focus of the included studies was examining the key SDoH (food insecurity, housing issues and social isolation) including psychological distress and well-being of these students, prompting the inclusion of broad search terms such as wellbeing, depression, anxiety, stress, and mental health. Table 2 represents the inclusion and exclusion criteria of this scoping review.

**Table 2:** Inclusion and exclusion criteria

Criteria	Inclusion	Exclusion
Target Population	University/higher education/tertiary students (both international and domestic) • Undergraduate students • Graduate students • Post graduate students	Other students: • Primary school students • Middle school students • High school students • Young adults in general • Adults in general
Comparison	International vs. domestic students	Only international students
Setting	Higher education institutions including university and college	Primary and secondary education institutes
Exposure	Student status (international students, domestic students)	Studies investigating all university student
Outcome	Investigate the key factors of SDoH- food insecurity, social isolation, housing issue, psychological distress shaping the experiences and overall wellbeing	Studies looking into physical health outcomes
Study Design	Qualitative, quantitative, and mixed-method primary studies	Case reports, editorials, opinion pieces, secondary review studies
Publication Year	January 2014 to May 2024	Before 2014
Language	English	Any other language

### Study selection

All potentially identified studies were imported to Covidence systematic review software, where duplicates were identified and removed. The screening process involved two stages: initially, one reviewer (NF) assessed titles and abstracts (*n* = 895), followed by a full-text review (*n* = 134) conducted independently by three reviewers (NF, TS, and GG) based on the inclusion and exclusion criteria. Any disparities in selection were resolved through consensus, with another reviewer (SM) mediating as needed. This rigorous selection process ensured that only studies providing valuable data around the key SDoH, such as food insecurity, housing issues, social isolation, psychological distress and how it affects overall experiences and wellbeing of international students compared to domestic students, were considered.

### Quality assessment

To assess the quality of the studies included in the review, we utilised the JBI critical appraisal tools, which offer checklists tailored to specific study design [39]. A Microsoft Excel spreadsheet was used to apply the tool’s criteria for cross-sectional, cohort, and qualitative studies [39]. Each study was evaluated against eight to eleven quality criteria, depending on the study design, covering aspects such as study reliability, methodological rigor, and transparency in reporting. A minimum score of 6 out of 8, 7 out of 10 and 8 out of 11 was required for inclusion, ensuring the retention of studies with moderate to high quality. While ethical approval was not a strict criterion, studies needed to demonstrate clear objectives, appropriate methodology, and robust data collection and analysis. This ensured a methodologically sound yet comprehensive evidence base aligned with the scoping review approach. The quality assessment was conducted by one first reviewer (NF) and verified by one of two secondary reviewers (TS or GG). Final decisions on the inclusion of the 23 studies were made collectively by NF, TS, and GG, ensuring all studies met the necessary quality criteria.

### Data extraction

For the data extraction, one author (NF) developed a preliminary data extraction table including all the relevant findings for this scoping review. The following data were extracted: study characteristics (title, year of publication, aim/objectives, country of origin and target population), study design, number of participants, outcome measures, and a description of the key findings related to the key SDoH (food insecurity, housing issue, social isolation and psychological distress) and its effect on wellbeing of international students in comparison to domestic students. Relevant data were extracted by one author (NF) and to check for accuracy and consistency, 20% of the extracted data were cross-checked by another author (TS).

### Synthesis and analysis of results

The findings were reported descriptively using tables with study characteristics. The information extracted from the chosen literature was organised according to the guidelines outlined in the PRISMA-ScR checklist for reporting results [36]. Data were analysed using a narrative synthesis approach [40]. To guide the reporting of the results, the SDoH were categorised into food insecurity, housing issues, social isolation, and psychological distress and wellbeing to examine how the experiences vary based on student status. This narrative synthesis approach synthesises findings from multiple studies by primarily using words and text to summarise and explain the results [40]. The data were summarised based on common key findings, similarities, differences, and gaps in the literature reviewed. Raw data were clustered to identify patterns and overarching categories. Studies with similar findings were reported alongside any studies with contrasting results and analysed. Any modifications were reviewed and revised by all researchers through an iterative process.

## Results

**Fig. 2:**
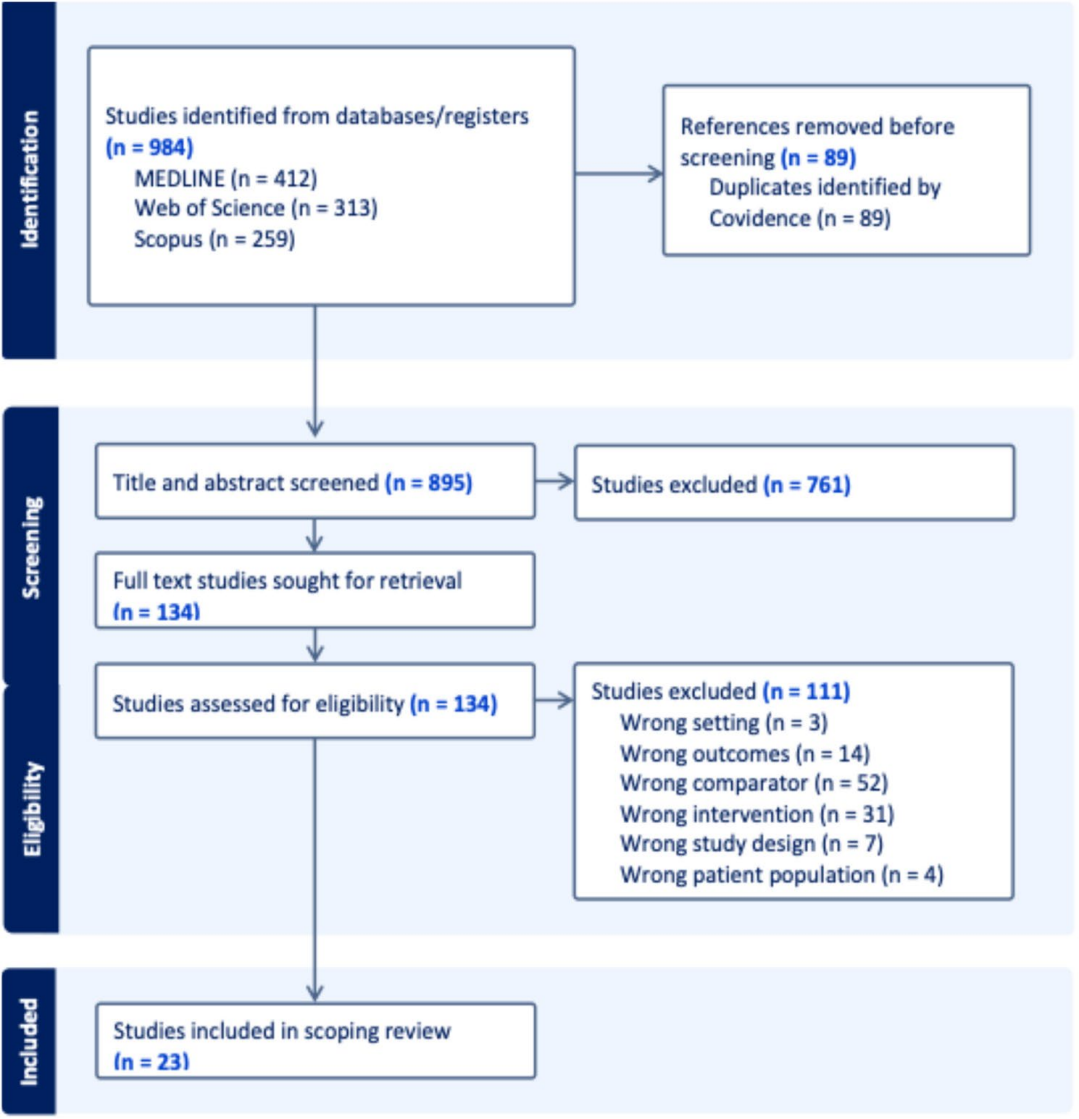
PRISMA Flow Diagram [41]

The three databases resulted in 984 studies, with 895 unique studies after removing 89 duplicates. The title and abstract screening stage excluded 761 studies using predefined inclusion and exclusion criteria. The remaining 134 studies were screened in full-text screening stage where 111 of these studies were deemed unsuitable for inclusion in the final review for various reasons, such as incorrect outcomes, interventions, comparators, or settings. A significant portion (*n* = 52) of these exclusions occurred because the studies only focused on international students without comparison to domestic students. A total of 23 peer-reviewed journal studies are included in this scoping review. The summary of this review process is outlined in Fig. 2.

### Quality assessment

The quality assessment of the included studies, summarised in Table 3, demonstrated high methodological rigor, with the majority scoring above the minimum inclusion threshold. These findings indicate that the studies included in this review are of sufficiently high quality, ensuring the reliability of the results.

**Table 3:** Quality appraisal using JBI critical appraisal tools

JBI critical appraisal checklist for Cross-sectional Studies												
Author(s) and Year	Q1	Q2	Q3	Q4	Q5	Q6	Q7	Q8	Total			
Prado et al., 2024 [42]	Y	Y	Y	Y	Y	Y	Y	Y	8			
Kivelä et al., 2022 [43]	Y	Y	Y	Y	Y	Y	Y	Y	8			
Amanvermez et al., 2023	Y	Y	Y	Y	Y	Y	Y	Y	8			
Bennett et al., 2022 [44]	Y	Y	Y	Y	Y	Y	Y	Y	8			
Dana et al., 2023 [45]	Y	Y	Y	Y	Y	Y	Y	Y	8			
Hanbazaza et al., 2017 [46]	Y	Y	Y	Y	Y	Y	Y	Y	8			
Yeung et al., 2022 [47]	Y	Y	Y	Y	U	U	Y	Y	6			
Mihrshahi et al., 2022 [23]	Y	Y	Y	Y	Y	Y	Y	Y	8			
Rekenyi et al., 2023 [48]	Y	Y	Y	Y	Y	Y	Y	Y	8			
Shi et al., 2023 [49]	Y	Y	Y	Y	Y	Y	Y	Y	8			
Skromanis et al., 2018 [10]	Y	Y	Y	Y	Y	Y	Y	Y	8			
Smith et al., 2022 [50]	Y	Y	Y	Y	Y	Y	Y	Y	8			
Larcombe et al., 2023 [51]	Y	Y	Y	Y	U	U	Y	Y	6			
Marczuk et al., 2023	Y	Y	Y	Y	Y	Y	Y	Y	8			
LaMontagne et al., 2023 [52]	Y	Y	Y	Y	Y	Y	Y	Y	8			
Kamardeen et al., 2018 [53]	Y	Y	Y	Y	N	N	Y	Y	6			
Rosa et al., 2023 [54]	Y	Y	Y	Y	Y	Y	Y	Y	8			
JBI critical appraisal checklist for Cohort Studies												
Author(s)	Q1	Q2	Q3	Q4	Q5	Q6	Q7	Q8	Q9	Q10	Q11	Total
Russell et al., 2023 [55]	Y	Y	Y	Y	Y	Y	Y	Y	Y	Y	Y	11
Dingle et al., 2024 [56]	Y	Y	Y	U	U	Y	Y	Y	Y	Y	Y	9
King et al., 2023 [57]	Y	Y	Y	Y	Y	Y	Y	Y	Y	Y	Y	11
Dingle et al., 2022 [58]	Y	Y	Y	U	U	Y	Y	Y	Y	Y	Y	9
JBI critical appraisal checklist for Qualitative Studies												
Author(s)	Q1	Q2	Q3	Q4	Q5	Q6	Q7	Q8	Q9	Q10	Total	
Wright et al., 2021 [59]	Y	Y	Y	Y	Y	N	N	Y	Y	Y	8	
Aydın 2020 [60]	Y	Y	Y	Y	Y	N	N	Y	U	Y	7	

### Study characteristics

Table 4 (below) shows the summary of study characteristics and Table 5 shows the study characteristics of all 23 included studies in details.

**Table 4:** Summary of study characteristics

Characteristics	Sub-category of Characteristics	Articles (*n* = 23)	
		**n**	**%**
Study Design	Quantitative	21	91%
	Qualitative	2	9%
Publication Year	2014–2019	3	13%
	2020–2022	8	35%
	2023–2024	12	52%
Study Location	United States	2	9%
	Australia	11	48%
	Canada	4	17%
	Germany	2	9%
	Netherlands	2	9%
	Others	2	8%
Sites	Single	16	70%
	Multiple	7	30%
Number of Participants	1–100	3	13%
	101–1000	7	30%
	1001–2000	4	18%
	2001–5000	4	17%
	> 5001	5	22%
	Total Number	132,341	-
	Range	31–44,851	-
Target Population	Majority International Students (> 60%)	3	13%
	Majority Domestic/Local Students (> 60%)	17	74%
	Mixed (Almost equal domestic/international)	3	13%
Age	18–25	12	52%
	26–40	3	13%
	40 and above	1	4%
	Not Specified	7	31%
Gender	Majority Female (> 60%)	14	52%
	Mixed (Almost equally)	4	22%
	Not Specified	5	26%
Associated Key Factors of SDoH (a study may include more than one factors)	Social Isolation	17	74%
	Food Insecurity	9	39%
	Housing Issues	5	22%
	Psychological Distress and Wellbeing	22	96%
	COVID-19 pandemic	6	26%
	Other (Academic and Financial Stressors)	9	39%

**Table 5:** Study characteristics

Author, Publication year	Setting (country)	Study design	Methodology and Data Collection Timeline	Target population	Sample size			Participant characteristics (age, gender)
					International Students	Domestic Students	All Students	
Quantitative Studies								
Prado et al., 2024 [42]	German universities (Europe - Germany)	Cross-sectional Study	Online surveys (in 2020, 2021, and 2022)	International and Domestic Students	*n* = 546	*n* = 13,952	*n* = 14,498	Mean Age: 23.72; Female: 69.53%; Male: 28.96%
Kivelä et al., 2022 [43]	Multiple universities (Europe - Netherlands)	Repeated Cross-sectional Study	Online survey study (in 2020, 2021)	Domestic (Dutch) and international students	March 2020 39%; March 2021 62%	March 2020 61%; March 2021 38%	March 2020 *n* = 207; March 2021 *n* = 142	Mean Age: 20–21; Female: 80–82%
Russell et al., 2023 [55]	A tertiary education institution in Melbourne (Australia)	Cohort study	Online health and well- being survey (in April/May 2019, September/October 2020)	University Students - local and international	*n* = 1245	*n* = 3162	Both waves: 4407 university students participated; wave 1: 14,880 students; wave 2: 9011 students	Mean Age: 23.4; Female: 68.3%; Male: 31.1%; Non-binary: 0.6%
Amanvermez et al., 2023	Two universities in Amsterdam (Europe - Netherlands)	Cross-sectional study	Survey; March 2018 and September 2019	College Students	*n* = 554	*n* = 1642	*n* = 2,196 college students	Age: 18 years old or older
Bennett et al., 2022 [44]	Australian public research university, Monash University, Melbourne (Australian)	Cross-sectional study	Online surveys; July and October 2020	Undergraduate and postgraduate university students	*n* = 318	*n* = 513	*n* = 1315 (484 didn’t respond about student status)	Mean Age: 23.2 to 24.5; Female: 41.9% to 55.6%
Dana et al., 2023 [45]	A university in Western Australia (Australia)	Cross-sectional study	Survey; 20 July and 25 September 2020	Students - undergraduate and postgraduate	*n* = 111	*n* = 102	*n* = 213	Mean Age: 26.07; Female: 70% (*n* = 149)
Dingle et al., 2024 [56]	University of Queensland, Australia	Longitudinal study	Online survey (Four cohort − 2019, 2020, 2021, 2022)	International and Domestic Students	2019 cohort: *n* = 179; 2020 cohort: *n* = 98; 2021 cohort: *n* = 99; 2022 cohort: *n* = 50	2019 cohort: *n* = 293; 2020 cohort: *n* = 301; 2021 cohort: *n* = 266; 2022 cohort: *n* = 251	*n* = 1540	Mean Age: 20.1; Female: 73.9%
Hanbazaza et al., 2017 [46]	University of Alberta (US)	Cross-sectional study	Interviewer-administered survey; April 2013 to April 2014	Student clients of the Ualberta campus food bank CFB	*n* = 27	*n* = 31	*n* = 58	-
Yeung et al., 2022	American College Health Association-National College Health Assessment (ACHA-NCHA) − 92 institutions (US)	Cross-sectional study	Paper survey or the web-based survey; twice a year in the fall and spring semesters	Degree-seeking undergraduate domestic and international students	*n* = 2,423	*n* = 42,428	*n* = 44,851	Age: 18 years old or older; Female: domestic (67.8%) and international (59.3%) student
King et al., 2023 [57]	Canadian university - Queen’s University (Canada)	Cohort study	Electronic/Online surveys; September 2018 and March 2019	Undergraduate students	Baseline *n* = 297; Follow-up *n* = 143	Baseline *n* = 2694; Follow-up *n* = 1794	Baseline *n* = 2991; Follow-up *n* = 1937	Int more likely to be older; Asian ethnicity, Families with lower parental education
Mihrshahi et al., 2022 [23]	Macquarie University (Australia)	Cross-sectional study	Online survey; July and September 2020	Students	*n* = 39 (37.1%)	*n* = 66 (62.9%)	*n* = 105	Age Range: 18–59 years; Female: 84.6% (88/105).
Rekenyi et al., 2023 [48]	Second largest university in Hungary (Europe - Germany)	Cross-sectional study	Online survey; May 2020, June 2020, July-Aug 2021 (at three-time intervals during the pandemic)	International and Hungarian students	May 2020: *n* = 341; June 2020: *n* = 104; July-Aug 2021: *n* = 34	May 2020: *n* = 948; June 2020: *n* = 142; July-Aug 2021: *n* = 105	May 2020: *n* = 1320; June 2020: *n* = 246; July-Aug 2021: *n* = 139	Female: 56.7% − 58.9% (Int); Female: 71.8% − 75.8% (Domes);
Shi Y and Allman-Farinelli M., 2023 [49]	A large Australian university in Sydney (Australia)	Cross-sectional study	Online survey (October 2021, May 2022)	Domestic and international students	*n* = 91	*n* = 376	*n* = 467	Age Range: 18–30; Female: 74.9%
Skromanis et al., 2018 [10]	University of Tasmania (Australia)	Cross sectional study	Online Survey (nested in a larger survey)	International and domestic undergraduate and postgraduate students	*n* = 382	*n* = 1013	*n* = 1395	Age Range: 18–72; Origin China, Malaysia, India and Singapore
Smith et al., 2022 [50]	Canadian University (Canada)	Cross sectional study	Survey	Domestic and international students entering university	*n* = 86	*n* = 185	*n* = 272	Age Range: 16–48; Female: 146; Male: 120
Dingle et al., 2022 [58]	A metropolitan university in Australia (Australia)	Longitudinal study	Survey	First-year students	*n* = 30.4%	*n* = 69.6%	*n* = 1239	2019 - Mean Age: 19.9; 2020 - Mean Age: 19.5; 2021 - Mean Age: 20.7; 2019 - Female: 69.3%; 2020 - Female: 73.9%; 2021 - Female: 76.2%
Larcombe, W. et al., 2023 [51]	Large, metropolitan Australian university	Cross sectional study	Survey	Bachelor, Master or research higher degree (RHD) students	*n* = 1609	*n* = 3794	*n* = 5403	Age Range: 21 and younger; Female: 60–63.5%, Male: 36.5–40%
Marczuk, A., & Lörz, M., 2023	23 Universities Nationwide	Cross sectional study	Survey	University students	*n* = 1424	*n* = 18,925	*n* = 20,349	-
LaMontagne, A.D., et al., 2023 [52]	A large Australian University	Cross Sectional Study	Survey	International and Domestic Students	*n* = 883	*n* = 2852	*n* = 3735	Female: 69%, Male: 31%
Kamardeen, I. and Sunindijo, R.Y., 2018 [53]	Australian University	Cross Sectional Study	Survey	International and Domestic Students	*n* = 32	*n* = 75	*n* = 107	-
Rosa D. et al., 2023 [54]	Canadian University	Cross Sectional Study	Survey	International and Domestic Students	*n* = 605	*n* = 4035	*n* = 4640	Mean age 22 years; 31–36% male; 64–69% female
Qualitative Studies								
Wright et al., 2021 [59]	University of Nevada, Reno (an urban, Western university) (US)	Qualitative Study	Semi-structured interviews (January– April 2020)	Second Generation Americans (SGA) and International (INT) students	*n* = 15	*n* = 16	*n* = 31	Age: 18 years or older; Female: 20; Male: 11
Aydın OT., 2020 [60]	Istanbul Bilgi University (Europe - Turkey)	Qualitative Design	Semi structured face-to-face interviews, lasting 30–40 min	International and Local students	*n* = 42	*n* = 35	*n* = 77	Int Student - Female: 22, Male: 20; Local Student - Female: 16, Local: 19

Among the 23 studies, 91% (*n* = 21) were quantitative, with the majority being cross-sectional (*n* = 17), including one repeated cross-sectional study [43]. The remaining studies included two longitudinal and two cohort designs [55–58]. Only two studies used qualitative study designs using semi-structured open-ended questionnaires [59, 60]. The study sample sizes ranged from the smallest sample of 31 participants in a qualitative study, comprising 16 domestic students and 15 international students, to the largest sample of 44,851 respondents in a cross-sectional study, including 42,428 domestic students and 2,423 international students [44, 59]. Within the review time frame of 2014 to 2024, there was a substantial increase in peer-reviewed publications from COVID-19 onwards where the majority were published between 2020 and 2024 (*n* = 20) while only 13% (*n* = 3) were published between 2014 and 2019. Among the 23 studies, 48% were conducted in Australia (*n* = 11), 26% in North America (the United States (*n* = 2) and Canada (*n* = 4), and 26% in Europe (Netherlands, Germany, Portugal, and Turkey). The majority (74%) had more domestic students, and 52% had participants aged 18 to 25. Gender was unspecified in 26% of the studies; of those that reported it, 52% had mostly female participants.

Across the 23 studies, a variety of tools were used to evaluate key SDoH, including eighteen instruments used for psychological distress, seven for wellbeing, three for food insecurity, eight for social isolation, and one for housing issues (Table 6 and Supplementary Table 1). Notable instruments used include the PHQ-9 [42, 57, 61], PSS-4, K10 [10, 23, 42, 57], and GAD-7 [57, 61] for distress, the WHO-5 and Ryff’s scales for wellbeing [44, 47, 49, 51, 56, 58], USDA tools for food insecurity [46], and the UCLA Loneliness Scale for social isolation [10, 42, 48, 58].

**Table 6:** Measurement scales used for assessing key SDoH & psychological distress and wellbeing (*n*=21)

**Author, Publication Year**	**Psychological Distress**																			
	**Depression**								**Stress**						**Anxiety**					
	**PHQ-9**	**PHQ-2**	**BDI-I**	**GMDS**	**PROMIS DA Scale**	**PC DASD**		**20-item PC Screen**	**PSS-4**	**PSS-10**	**MIDUS**	**DASS-21**	**K10**		**BAI**	**GAD-2**	**GAD-7**	**BSSI**	**DSM-V PTSD**	**LSPSS**
Prado et. al., 2024 [42]	x								x											
Kivelä et. al., 2022 [43]			x												x			x	x	x
Russell et. al., 2023 [55]		x														x				
Amanvermez et. al., 2023	x										x						x			
Bennett et. al., 2022 [44]					x					x										
Dana et. al., 2023 [45]												x								
Dingle et. al., 2024 [56]						x		x												
Hanbazaza et. al., 2017 [46]																				
Yeung et. al., 2022																				
King et. al., 2023 [57]	x								x								x			
Mihrshahi et. al., 2022 [23]													x							
Rekenyi et. al., 2023 [48]			x	x																
Shi Y. & Allman-F. M., 2023 [49]																				
Skromanis et. al., 2018 [10]													x							
Smith et. al., 2022 [50]									x											
Dingle et. al., 2022 [58]																				
Larcombe, W. et. al., 2023 [51]												x								
Marczuk, A., & Lörz, M., 2023																				
LaMontagne, A.D., et. al., 2023 [52]																				
Kamardeen, I. and Sunindijo, R.Y., 2018 [53]												x								
Rosa D. et. al., 2023 [54]								x												

**Author, Publication Year**	**Psychological Wellbeing**								**Food Insecurity**			**Social Isolation and Social Support**								**Housing Issues**
	**WHO PWI**	**Short WEMWS**	**MH Screening**	**CSSWQ Subscale**	**SLS**	**Ryff Scale PWB**	**Self-Rated**		**USDA HFSSM – 6 items**	**USDA HFSSM – 10 items**	** USDA HFSSM – 18 items**	**UCLA 3 - Loneliness Scale**	**DJGLS**	**PROMIS SI**	**5-item ESSI**	**MOS-SSS-6**	**SRS**	**PROMIS ES**	**MSPSS**	**WHOQOL-BREF**
Prado et. al., 2024 [42]												x			x					
Kivelä et. al., 2022 [43]													x							
Russell et. al., 2023 [55]																x				
Amanvermez et. al., 2023																				
Bennett et. al., 2022 [44]	x								x					x				x		
Dana et. al., 2023 [45]											x									
Dingle et. al., 2024 [56]		x	x																	
Hanbazaza et. al., 2017 [46]							x			x										
Yeung et. al., 2022							x													
King et. al., 2023 [57]				x																
Mihrshahi et. al., 2022 [23]									x											
Rekenyi et. al., 2023 [48]																			x	
Shi Y. & Allman-F. M., 2023 [49]	x						x				x									
Skromanis et. al., 2018 [10]					x														x	x
Smith et. al., 2022 [50]							x										x			
Dingle et. al., 2022 [58]		x					x					x								
Larcombe, W. et. al., 2023 [51]						x														
Marczuk, A., & Lörz, M., 2023														x						
LaMontagne, A.D., et. al., 2023 [52]							x													
Kamardeen, I. and Sunindijo, R.Y., 2018 [53]																				
Rosa D. et. al., 2023 [54]						x														

*Outcome Measures- PHQ *Patient Health Questionnaire, *BDI *Beck Depression Inventory, *GMDS *Gotland Male Depression Scale, * PROMIS *Patient-Reported Outcomes Measurement Information System, *PC *PsyCheck,*DA *Depression and Anxiety,*DASD *Depression, Anxiety and Somatic Distress *PSS *Perceived Stress Scale, *MIDUS *Midlife Development in the U. S.,*DASS *Depression, Anxiety, and Stress Scale, *K10 *Kessler psychological distress scale, *BAI *Beck Anxiety Index, *BSSI *Beck Scale for Suicide Ideation, *DSM-V PTSD *PTSD Checklist for DSM-V (PCL-5), * LSPSS *Law Student Perceived Stress Scale, *WHO PWI *World Health Organization Psychological Wellbeing Index, *WEMWS *Warwick–Edinburgh Mental Wellbeing Scale, * MH *Mental Health, *CSSWQ *College Student Subjective Wellbeing Questionnaire,*SLS *Satisfaction with Life Scale, *USDA HFSSM *Household Food Security Survey Module, *DJGLS *De Jong-Gierveld Loneliness Scale, *PROMIS SI* Patient-Reported Outcomes Measurement Information System SocialIsolation, *5-item ESSI*Five-item ENRICHED Social Support Inventory, *MOS-SSS-6 *MOS Social Support Survey 6 item, *SRS *Social Relationships Scale, *PROMIS ES *Patient-Reported Outcomes Measurement Information System Emotional Support, *MSPSS *Multidimensional Scale of Perceived Social Support, *WHOQOL-BREF *Environmental Health subscale of the World Health Organization Quality of Life Short Form

Based on the conceptual framework presented in Fig. 1 above, the summary statistics from the 21 quantitative studies included in this review are summarized in Tables 7 and 8.

**Table 7:** Summary statistics of structural determinants and social cohesion and social capital

Author, Publication year	Structural Determinants: Social Determinants of Health (SDoH)					Social Cohesion and Social Capital					
	Student Status	Context/Time Points	Others: Education/Academic Stressors, Pandemic Stressors and Income/Financial Stressors			Social Support			Loneliness		
			M(SD)/Prevelance (CI)	Effect of Student Status (SS) and Timepoints (TP)		M(SD)/Prevelance (CI)	Effect of Student Status (SS) and Timepoints (TP)		M(SD)/Prevelance (CI)	Effect of Student Status (SS) and Timepoints (TP)	
				F-test/χ2-test/Odds Ratio/Cohen’s d/Adjusted RR/𝛃 Coefficient	P-value		F-test/χ2-test/Odds Ratio/Cohen’s d/Adjusted 𝛃 Coefficient	P-value		F-test/χ2-test/Odds Ratio/Cohen’s d/Adjusted 𝛃 Coefficient	P-value
Prado et al., 2024 [42]	International Students *n* = 546	2020, 2021, 2022				18.38 (5.72)	SS: F(1, 14489) = 300.373; TP: F(2, 14489) = 6.746	*p* < 0.001	4.39 (1.74)	SS: F(2, 14489) = 0.759; TP: F(2, 14489) = 156.706	SS: *p* = 0.384; TP: *p* < 0.000
	Domestic Students *n* = 13,952					21.52 (3.54)			4.55 (1.58)		
Kivelä et al., 2022 [43]	International Students *n* = 169	2020, 2021	Academic: 54.2 (10.2)	SS: 19.6; TP: 11.3	SS: *p* < 0.001; TP: *p* = 0.001				5.67 (3.31)	SS: 40.7; TP: 13.8	*p* < 0.001
	Domestic Students *n* = 180		Academic: 49.1 (9.50)						3.02 (3.15)		
Russell et al., 2023 [55]	International Students *n* = 1245	Wave 1 and 2 (pre and during pandemic)	Pandemic: 860 (71.8)			63.9 (61.0, 66.7) − 68.0 (65.4, 70.7)	OR 2.64	*p* < 0.001			
	Domestic Students *n* = 3162		Pandemic: 2181 (70.5)			37.6 (35.9, 39.4) − 37.9 (36.2, 39.6)					
Amanvermez et al., 2023	International Students *n* = 554	Mar 2018 - Sept 2019	Financial: 0.31 (0.07)	F(1, 2195) = 20.86	*p* < 0.001						
	Domestic Students *n* = 1642										
Bennett et al., 2022 [44]	International Students *n* = 318	2020									
	Domestic Students (*n* = 513)										
Dana et al., 2023 [45]	International Students *n* = 111	2020									
	Domestic Students *n* = 102										
Dingle et al., 2024 [56]	International Students *n* = 426	2019, 2020, 2021, 2022									
	Domestic Students *n* = 1111										
Hanbazaza et al., 2017 [46]	International Students *n* = 27	2014									
	Domestic Students *n* = 31										
Yeung et al., 2022	International Students *n* = 2,423	2017									
	Domestic Students *n* = 42,428										
King et al., 2023 [57]	International Students *n* = 297 and 143	Baseline and Follow up				Social Competence: Male: 13.3 (4.0); Female: 13.7 (4.4); Social support: Male: 15.3 (3.8); Female: 16.1 (3.9);	Social Competence: Male: Cohen’s d = 0.02; *p* = 0.91; Female: Cohen’s d = 0.02, *p* = 0.90; Social Support: Male: Cohen’s d = 0.10; *p* = 0.34; Female: Cohen’s d = 0.13, *p* = 0.14				
	Domestic Students *n* = 2694 and 1794					Social Competence: Male: 13.2 (4.6); Female: 13.6 (4.2); Social Support: Male: 15.7 (4.5); Female: 16.6 (3.8);					
Mihrshahi et al., 2022 [23]	International Students *n* = 39	2020									
	Domestic Students *n* = 66										
Rekenyi et al., 2023 [48]	International Students *n* = 341, 104, 34	April 2020, June 2021, July 2021				M = 63.89% (female higher median (62) than the male students (55))	Male: −0.208, Female: −0.004	*p* < 0.001			
	Domestic Students *n* = 948, 142, 105					M = 80.00% (Female higher values (M = 45) than male students (M = 41))	Male: −0.219, Female: −0.186	*p* < 0.001			
Shi Y and Allman-Farinelli M., 2023 [49]	International Students *n* = 91	Oct 2021 - May 2022									
	Domestic Students *n* = 376		Academic: 240 (51.4)	Financial: OR = 2.68; 95% CI, 1.40–5.13	*p* < 0.001						
Skromanis et al., 2018 [10]	International Students *n* = 382	-				Significant Others: 3.6 (1.2); Family: 3.9 (0.9); Friends: 3.7 (1.0)	t = 6.0, t = 1.9, t = 3.0	*p* < 0.01, *p* = 0.05, *p* < 0.01			
	Domestic Students *n* = 1013					Significant Others: 4.0 (1.2); Family: 4.0 (1.0); Friends: 3.9 (1.1)					
Smith et al., 2022 [50]	International Students *n* = 86	-				Rank Avg: 156.03	U = 8.749	*p* = 0.003			
	Domestic Students *n* = 185					Rank Avg: 126.69					
Dingle et al., 2022 [58]	International Students *n* = 378	2019, 2020, 2021				2019: 3.47 (0.96); 2020: 3.22 (1.08); 2021: 3.39 (0.93)		TP: 2019–2020: *p* = 0.004; 2020–2021: *p* = 0.015	2019: 5.83 (1.78); 2020: 6.25 (1.78); 2021: 4.77 (1.63),	SS: F(1) = 5.96; TP: F(2) = 58.90	SS: *p* = 0.015; TP: *p* < 0.001
	Domestic Students *n* = 861										
Larcombe, W. et al., 2023 [51]	International Students *n* = 1609	2017–2018									
	Domestic Students *n* = 3794										
Marczuk, A., & Lörz, M., 2023	International Students *n* = 1424	2020	Academic: Delay: 56%; Coping 0.53; Financial: Hardship 0.58; Loss of job 0.27; Worse income 0.18; Reduced support 0.13, job loss 0.29		Academic: Delay *p* < 0.001; Coping *p* < 0.001; financial hardship *p* < 0.001; loss of job *p* < 0.01; Worsening income *p* < 0.001; reduced support *p* < 0.001, job loss *p* < 0.001	SS: Contact with students: 0.87; Participation in groupwork: 0.77; TP: communication with lecturers 0.15 and reduced contact with other students 0.07		Contact with students: *p* < 0.001; Participation in groupwork: *p* < 0.05; TP: communication with lecturers *p* < 0.001 and reduced contact with other students *p* < 0.001			
	Domestic Students *n* = 18,925		Academic: Delay 46%; Coping 0.64; Financial hardship 0.30; Loss of job 0.23; Financial hardship 0.30; Loss of job 0.23			Contact with students: 0.81; Participation in groupwork 0.76					
LaMontagne, A.D., et al., 2023 [52]	International Students *n* = 883	July, 2019									
	Domestic Students *n* = 2852										
Kamardeen, I. and Sunindijo, R.Y., 2018 [53]	International Students *n* = 32	August 2016 - May 2017	Financial: 2.34		*p* = 0.398	Family Support 1.75; Lack of support 1.45		*p* = 0.041*; *p* = 0.434	2.25		*p* = 0.000*
	Domestic Students *n* = 75		Financial: 2.19			Family Support 2.23; Lack of support 1.34			1.49		
Rosa D. et al., 2023 [54]	International Students *n* = 605	-									
	Domestic Students *n* = 4035										

**Table 8:** Summary statistics of intermediary determinants

**Author, Publication year **	**Student Status**	**Intermediary Determinants: Social Determinants of Health (SDoH)**									
		**Food Insecurity**			**Housing Issues**				**Psychological Factors: Depressive Symptoms**		
		M(SD)/ Prevelance (CI)	Effect of Student Status (SS) and Timepoints (TP)		M(SD)/ Prevelance (CI)	Effect of Student Status (SS) and Timepoints (TP)			M(SD)/ Prevelance (CI)	Effect of Student Status (SS) and Timepoints (TP)	
			F-test/χ2-test/Odds Ratio/Cohen's d/Adjusted 𝛃 Coefficient	*P*-value		F-test/χ2-test/Odds Ratio/Cohen's d/Adjusted 𝛃 Coefficient	*P*-value			F-test/χ2-test/Odds Ratio/Cohen's d/Adjusted 𝛃 Coefficient	*P*-value
Prado et. al., 2024 [42]	Int. *n* = 546								10.99 (6.41)	SS: F(1, 14489) = 64.216; TP: F(2, 14489) = 13.559	*p* <0.001
	Domes. *n* = 13,952								8.57 (5.39)		
Kivelä et. al., 2022 [43]	Int. *n* = 169								14.1 (10.0)	SS: 13.1; TP: 24.2	*p* <.001
	Domes. *n* = 180								9.73 (8.26)		
Russell et. al., 2023 [55]	Int. *n*=1245	7.9 (6.4, 9.6) - 16.3 (14.3, 18.5)	OR 5.21	*p* < 0.001					22.8 (20.4, 25.4) - 42.6 (40.0, 45.4)	OR 1.43	*p* < 0.001
	Domes. *n*=3162	10.0 (8.9, 11.2) - 4.3 (3.7, 5.1)							21.7 (20.4, 23.0) - 34.4 (32.8, 36.1)		
Amanvermez et. al., 2023	Int. *n*=554								7.43 (0.30)	Cohen's d −0.41	*p* < .001
	Domes. *n*=1642								5.37 (0.13)		
Bennett et. al., 2022 [44]	Int. *n*=318	52.4 (33)		*p* <0.001							
	Domes. *n*=513	17.5 (11)							65.0 (10.3)	FI: 7.2 (4.1, 10.3)	*p* <0.001
Dana et. al., 2023 [45]	Int. *n*=111	48%; With: 21 (38); Without: 81 (52)	AOR 9.13; 95% CI: 2.32–35.97	*p* = 0.002; With: χ2 28.39, p ˂0.001; Without: χ2 49.91; p ˂0.001;					14.81 (11.49)	SS: 12.11; FI: AOR 1.62; 95% CI: 1.12–2.33	SS: *p* <0.001; FI: *p* = 0.010
	Domes. *n*=102	With: 35 (62); Without: 74 (48);									
Dingle et. al., 2024 [56]	Int. *n*=426								1.0 (1.3)	F(3, 1494) = 3.60	*p* = 0.013
	Domes. *n*=1111								1.4 (1.4)		
Hanbazaza et. al., 2017 [46]	Int. *n*=27	Marginal (14.8%), moderate (37.0%), or severe (44.4%) - 96.2%									
	Domes. *n*=31	Marginal (3.2%), moderate (51.6%), or severe (45.2%) - 100%									
Yeung et. al., 2022	Int. *n*=2,423									OR 0.84 (CI: 0.66–1.06)	Not significant
	Domes. *n*=42,428										
King et. al., 2023 [57]	Int. *n*=297 and 143									SS: Male: β=0.40 (SE 0.21); Female: β=−0.02 (SE 0.18) ; TP: Male: β=0.61 (SE 0.10); Female: β=0.56 (SE0.06)	SS: Male: *p* =0.06; *p* = 0.93; TP: Male: *p*<.01; *p* <.01
	Domes. *n*=2694 and 1794										
Mihrshahi et. al., 2022 [23]	Int. *n*=39	29 (74.4%)							27.13, (9.71)	SS: t(90) = 2.68; FI: OR = 8.07 (95% CI 3.0–21.8)	SS: *p* = 0.009; FI: *p* < 0.001.
	Domes. *n*=66	15 (22.7%)							22.08 (7.85)		
Rekenyi et. al., 2023 [48]	Int. *n*=341, 104, 34								Male Depression Score: 13; Female Depression Score: 17		*p* <0.001
	Domes. *n*=948, 142, 105								Male Depression Score: 13; Female Depression Score: 11		
Shi Y and Allman-Farinelli M., 2023 [49]	Int. *n*=91	18.7% (95% CI, 0.2%–37.2%)	Adjusted OR = 2.02; 95% CI, 1.01–4.07 (Academic and Financial)	*P* = 0.280; Adjusted OR: *P* = 0.049		Living arrangement changes: OR = 3.50; 95% CI, 1.15–10.59	*P* = 0.027				
	Domes. *n*=376	13.0% (95% CI, 3.6%–22.5%)				Accommodations other than the parental home: OR = 3.86; 95% CI, 2.02–7.37	*P* < 0.001				
Skromanis et. al., 2018 [10]	Int. *n*=382								20.6 (8.7)	t = 0.7	*p*=0.51
	Domes. *n*=1013								20.9 (8.3)		
Smith et. al., 2022 [50]	Int. *n*=86	Rank Avg: 121.13	U = 5.047	*p*=0.025	Rank Avg: 115.33	U = 9.860	*p*=0.002				
	Domes. *n*=185	Rank Avg: 142.91			Rank Avg: 145.61						
Dingle et. al., 2022 [58]	Int. *n*=378									0.468	*p* < .001
	Domes. *n*=861										
Larcombe, W. et. al., 2023 [51]	Int. *n*=1609								SS: 10.27 (9.55); FI: 9.60 (8.13)	RR 0.82 (CI: 0.71 - 0.93)	*p* = .003
	Domes. *n*=3794								SS: 11.67 (10.04); FI: 9.39 (8.71)		
Marczuk, A., & Lörz, M., 2023	Int. *n*=1424										
	Domes. *n*=18925										
LaMontagne, A.D., et. al., 2023 [52]	Int. *n*=883								74.2	OR = 0.479 (CI: 0.356, 0.643)	*p* < 0.001
	Domes. *n*=2852								85.9		
Kamardeen, I. and Sunindijo, R.Y., 2018 [53]	Int. *n*=32								4.22		*p* = 0.724
	Domes. *n*=75								4.53		
Rosa D. et. al., 2023 [54]	Int. *n*=605								b = - .1871 (SE = .0618), 95% CI = - .3082 to .0660		p statistically significant
	Domes. *n*=4035								b = .1865 (SE = .1363), 95% CI = - .0807–.4536		

**Author, Publication year **	**Intermediary Determinants: Social Determinants of Health (SDoH)**										
	**Psychological Factors: Stress and Anxiety**				**Psychological Factors: Other Mental Disorder and PTSD**				**Psychological Factors: Wellbeing**		
	M(SD)/ Prevelance (CI)	Effect of Student Status (SS) and Timepoints (TP)			M(SD)/ Prevelance (CI)	Effect of Student Status (SS) and Timepoints (TP)			M(SD)/ Prevelance (CI)	Effect of Student Status (SS) and Timepoints (TP)	
		F-test/χ2-test/Odds Ratio/Cohen's d/Adjusted 𝛃 Coefficient	*P*-value			F-test/χ2-test/Odds Ratio/Cohen's d/Adjusted 𝛃 Coefficient		*P*-value		F-test/χ2-test/Odds Ratio/Cohen's d/Adjusted 𝛃 Coefficient	*P*-value
Prado et. al., 2024 [42]	8.22 (2.78)	SS: F(1, 14432) = 39.597; TP: F(2, 14432) = 3.323	SS: *p*<0.001; TP: *p* = 0.036								
	7.31 (3.18)										
Kivelä et. al., 2022 [43]	17.4 (12.7)	SS: 14.6; TP: 7.43	SS: *p* <.001; TP: *p*=0.007		24.6 (18.0)	SS: 17.2; TP: 3.67		SS: *p* <.001; TP: *p* = 0.056			
	12.6 (9.77)				15.9 (13.9)						
Russell et. al., 2023 [55]	27.7 (25.1, 30.4) - 46.9 (44.1, 49.7)	OR 1.09	*p* = 0.26								
	34.1 (32.4, 35.9) - 47.0 (45.3, 48.8)										
Amanvermez et. al., 2023	5.95 (0.25)										
	4.48 (0.11)	−0.34	*p* < .001								
Bennett et. al., 2022 [44]											
	Anxiety: 67.8 (9.5); Stress: 25.4 (5.8)	Anxiety: 7.4 (4.5, 10.4); Stress: 4.2 (2.1, 6.3)	*p* <0.001						33.3 (21.8)	FI: −10.6 (−17.2, −4.0)	*p* = 0.002
Dana et. al., 2023 [45]	Anxiety: 10.53 (9.70); Stress: 15.27 (10.70)	Anxiety: 8.53; Stress: 3.06	Anxiety: *p* <0.001; Stress: 0.029								
Dingle et. al., 2024 [56]	1.2 (1.2)	F(3, 1492) = 2.75	*p* = 0.041						21.6 (4.1)	SS: F(1, 1491) = 0.21; TP: F(3, 1491) = 0.95	SS: *p* = 0.649; TP: *p* = 0.414
	1.8 (1.4)								21.4 (4.0)		
Hanbazaza et. al., 2017 [46]										14.8% vs 38.7%, X = 4.125	P= 0.04
Yeung et. al., 2022		Anxiety- OR 0.47 (CI: 0.38–0.58); Depression and Anxiety- OR 0.58 (CI: 0.50–0.67)	*p* < .001 ; *p* < .001			Other MH issue- OR 0.72 (CI: 0.59–0.87);		*p* <. 01			
King et. al., 2023 [57]	Perceived Stress: Male: 6.8 (2.6); Female: 6.8 (2.3)	SS: Male: β=0.14 (SE 0.26); Female: β=−0.57 (SE 0.21); Perceived Stress: Male Cohen’s d = 0.35; Female Cohen’s d = 0.13; TP: Male: β=0.44 (SE 0.11); Female: β=0.41 (SE 0.07)	SS: Male: *p* =0.59; Female: *p* <.01; Perceived Stress: Male *p* <.01, Female *p* = .07; TP: Male: *p*<.01; *p* <.01								
	Perceived Stress: Male: 5.9 (2.5); Female: 7.2 (2.7)										
Mihrshahi et. al., 2022 [23]	23.73 (SD = 8.78; 95% CI 21.91–25.55)				11 (39.3%)						
Rekenyi et. al., 2023 [48]											
Shi Y and Allman-Farinelli M., 2023 [49]									11.88 ± 5.70	FI: OR = 2.98; 95% CI, 1.01–8.78 (poor wellbeing)	*P* = 0.048
									10.73 ± 5.28	FI: OR = 1.94; 95% CI, 1.06–3.57 (poor wellbeing)	*P* = 0.032
Skromanis et. al., 2018 [10]											
Smith et. al., 2022 [50]											
Dingle et. al., 2022 [58]		0.348	*p* < .001							TP: −.348	*p* < .001
Larcombe, W. et. al., 2023 [51]		Correlation with Depression 0.7; RR 1.03 (CI: 0.93 - 1.14)	*p* = 0.60						4.97 (0.71)	Correlation with Depression −.61; Correlation with Anxiety −.46; RR 1.14 (CI: 1.00 - 1.30)	*p* = 0.04
		Correlation with Depression 0.64;							5.07 (0.74)	Correlation with Depression −.67; Correlation with Anxiety −.47	
Marczuk, A., & Lörz, M., 2023											
LaMontagne, A.D., et. al., 2023 [52]					94.2	OR = 0.706 (CI: 0.403, 1.236)		*p* = not significant			
					95.6						
Kamardeen, I. and Sunindijo, R.Y., 2018 [53]	Anxiety 3.72 ; Stress: 5.41		*p* = 0.852, *p* = 0.081								
	Anxiety 3.56 ; Stress 7.16										
Rosa D. et. al., 2023 [54]	3.39 (.97)	F 75.72; b = 0.3736	*p* < .001						3.04 (1.27)	SS: F 9.41; TP: R2 = .148, F (5,4,415) = 19.59	*p* < .001
	3.72 (.81)								3.21 (1.21)		

### Vulnerabilities and triggers: key SDoH of international vs. domestic students

**Fig. 3:**
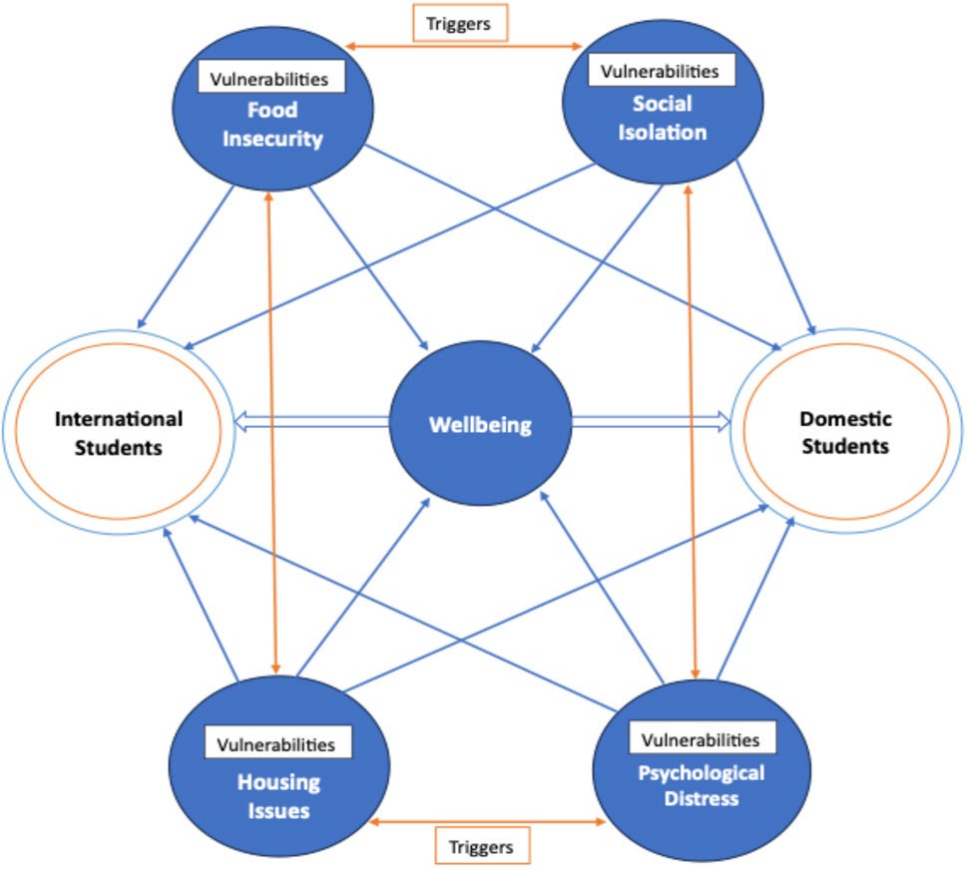
Complex interplay within key social determinants of health

Figure 3: Complex Interplay between Key Social Determinants of Health (SDoH) of International vs. Domestic Students. The figure illustrates how key SDoH food insecurity, housing issues, social isolation, and psychological distress affect students’ wellbeing both individually and collectively. The blue arrows show how each SDoH - food insecurity, housing issues, social isolation, and psychological distress - individually affects students’ wellbeing experiences while also collectively influencing it, with variations based on student status. These factors are highlighted as vulnerabilities. The orange arrows illustrate how the SDoH interact with and amplify one another, acting as additional triggers that intensify their overall impact on wellbeing.

Figure 3 provides an overview of the shared vulnerabilities and the interconnectedness of key social determinants of health for international and domestic students. While the figure does not visually differentiate the extent of impact between the two groups, it conceptually highlights the shared and interconnected vulnerabilities. Specific variations in the impact of these SDoH on international and domestic students are detailed in the text. The key findings of this review are summarised in Supplementary Table 2 [see additional file 2].

### Social isolation

Of the 23 studies included in this scoping review, 17 studies investigated social isolation and its effects on the experiences of university students based on their student status. A limited number of studies (*n* = 3) indicate that both domestic and international students experience significant social isolation and loneliness, with international students being more severely impacted than their domestic counterparts. For example, Prado et al. 2024 reported increasing loneliness over time among international students compared to domestic students [42, 46, 59, 61] and that there was a significant difference between social support received by international students in comparison to domestic students [42]. Another repeated cross-sectional study found that the odds of receiving social support were significantly greater among domestic students compared to international students, with an odds ratio of 2.64 (*p* < 0.05) [43]. Some studies, however, report no statistically significant differences in the prevalence of loneliness between domestic and international students [42, 58]. Conversely, multiple studies highlight that international students faced higher levels of isolation and loneliness due to cultural adjustments, lack of social support, and unfamiliarity with new environments, particularly during the COVID-19 pandemic [42, 43, 46–48, 53, 56, 57]. According to Kivela et al. (2024), 25% of international students experience severe loneliness, compared to 10% of domestic students [43].

Two studies showed that international students experience heightened social isolation and food insecurity due to limited social support network, which also contributes to worsened peer connections and housing difficulties [43, 55]. The key social support networks for both groups of students are significant others, family and friends. There is a statistically significant difference between international and domestic students in terms of support from significant others and friends, but both groups of students receive similar support from family members [10]. Compared to domestic students, international students have higher expectations for campus engagement to build social networks but often struggle to engage due to financial pressures and academic stress [50, 57]. Two of the included studies in this review reflect on gendered experiences of these two groups of students where according to one study, for social competence and social support, there was not a statistically significant difference between the men and women [57].

Various coping mechanisms were identified among students to mitigate social isolation. Unlike international students, domestic students tended to rely more on existing social networks such as family [23, 53]. International students exhibit greater self-efficacy and resilience in coping with loneliness despite having lower social support compared to domestic students [42]. Additionally, the lack of eligibility for government social support schemes leaves international students more vulnerable [49]. The necessity for tailored coping strategies was emphasised by a cohort study conducted in Australia, which pointed out the role of institutional support in supporting international students adjust [55].

### Food insecurity

Nine of the 23 included studies specifically focused on food insecurity, analysing different experiences based on student status. The findings highlighted significant food insecurity among students, with international students being particularly vulnerable [44, 55]. Across studies, the prevalence of international students living in food insecurity range from 48.3% to 83.4% where they face barriers around affordability of food, availability food, accessibility to shops and food bank, lack of food assistance program, finding culturally specific food, students with children, living arrangements, loss of casual employments etc [23, 44–46, 49, 50, 55, 59]. Bennett et al. (2022) reported that 18% of students experienced food insecurity, with 52.4% of those affected being international students [44]. During the pandemic, international students suffered from food insecurity to a greater extent compared to domestic students. Russell et al. (2023) indicated that international students experienced a five-fold increase in the odds of being unable to afford food during this period and they were at the more severe end of the spectrum [55]. The comparative analysis by Hanbazaza et al. (2017) and Dana et al. (2023) revealed that international students consistently faced significantly higher rates of low and very low food security compared to domestic students (90% vs. 20%) [44–46]. This disparity was even more pronounced among international students with children [45]. Overall, the findings illustrate the severe and pervasive nature of food insecurity among international students relative to their domestic counterparts. Some of the included studies report statistically significant differences, supported by adjusted odds ratios, confirming that international students are disproportionately impacted by food insecurity [45, 49, 50].

Additionally, financial stability is closely linked to food insecurity [46]. While domestic students primarily rely on government loans and jobs, international students depend on university funding and casual jobs to meet their daily food needs [46]. Research reveals that domestic students who lose their source of income are more likely to be food insecure; however international students who have unstable housing are more likely to be food insecure [49]. Social connections also play a role, with international students receiving less peer support for food compared to domestic students [46]. Beyond general food insecurity, cultural food insecurity coupled with social isolation from the absence of family mealtimes leads to identity loss and disconnection, adding further stress for international students [59].

The included studies found various coping mechanisms employed by students experiencing food insecurity. Dana et al. (2023) reported that both domestic and international students resorted to purchasing university supplies and accessing food-relief programs, although these measures were insufficient to address the underlying need of nutritional and healthy food [45]. Hanbazaza et al. (2017) highlighted the use of research funds and limited financial support as coping strategies among international students, who often lacked other sources of assistance [46].

### Housing issues

Of the 23 studies reviewed, only five discussed housing issues. International students faced more severe housing challenges due to their unfamiliarity with the local housing market and lack of a support network to secure stable, suitable and affordable housing [46, 50, 55, 56]. Most international students have unstable housing situations, living away from home in student or shared accommodations [56]. During the COVID-19 pandemic, their peer connections worsened, and they faced increased housing difficulties [55]. However, domestic students generally had better access to housing resources and support networks as already discussed in the previous section, often either living with families or relying on familial connections to secure stable living arrangements [49, 50, 56]. Here, there is a significant difference between the experiences of international students compared to domestic students [49].

One study reveals that domestic students living outside their parental home faced a higher risk of food insecurity because of reduced social support networks [49]. On the other hand, international students living in accommodations with provided meals, had higher odds of food insecurity compared to those in accommodations without meal provisions [49]. This study found that the association was attributed to the quality of meals provided, as these were often not tailored to meet the cultural and dietary preferences of international students [49]. Another study has revealed that international students are more concerned of access to food, safe place to live and importance of social network compared to domestic students [50]. These compounded effects create a complex web of stressors that significantly affect the experiences of university students disproportionately.

### Psychological distress

Psychological distress has become a significant issue among university students, with rising reports of depression, anxiety, stress and other mental health concerns, particularly during and after the COVID-19 pandemic [42, 51–54]. Of the 23 studies, 16 of the studies discussed about the depressive symptoms with majority reporting that international students exhibited higher mean levels and prevalence of depressive symptoms compared to domestic students [10, 23, 42, 43, 48, 51–55, 61]. The comparative statistics from the studies reflect that there is a statistically significant difference; Odds Ratio ranging from 0.479 to 13.1; between the experiences of depressive symptoms among international students in comparison to domestic students [10, 23, 42, 43, 48, 51–55, 61]. Only a few studies showed similar levels of depressive symptoms between the two groups with no statistically significant difference [10, 53]. Similarly, most of these studies also analysed level of stress and anxiety among these two groups of students and conducted a comparative analysis of the exposure among international and domestic students where three studies reflected on F statistics [42, 54, 56] and, rest on odds ratio [43, 45, 47, 55]. The sudden transition to remote learning, loss of routine, and reduced social interactions have all contributed to worsening mental health, with international students experiencing the greatest impacts. In 2021, both international and domestic students reported the highest levels of depressive symptoms, with increased rates of suicidal thoughts, particularly among international students [42, 47, 56]. Studies have shown that 9% of domestic students experienced moderate-to-severe depressive symptoms, 12% had probable PTSD, and 9% reported severe anxiety [43]. In comparison, international students were 1.5 times more likely to report an increase in depression and anxiety since the pandemic, with 20% experiencing moderate-to-severe depressive symptoms, 30% having probable PTSD, and 25% reporting severe anxiety [55].

In some of these studies, the psychological wellbeing has been analysed to see the correlation between wellbeing and other SDoH such as depression, anxiety, and food security to observe the experiences of international and domestic students. A study shows that there is no significant difference in the levels of anxiety and depression experienced by domestic and international students [51]. However, for both groups, there is a negative correlation between anxiety and depression and their overall wellbeing [51]. Students suffering from anxiety and depression being less likely to engage in social activities, leading to diminished social support networks. International students face greater isolation due to the added challenges of adjusting to a new cultural environment and navigating life far from home, which intensifies their mental health struggles [51, 53, 54, 62]. Food insecurity among international students has affected psychological distress, with deteriorating food security linked to higher depression and stress [44]. Those international students experiencing very low food security report higher levels of depression and anxiety compared to their food-secure peers and their wellbeing is affected [45, 54].

Students employed various coping mechanisms to manage their psychological distress and improve their wellbeing. Domestic students often turned to family and friends for emotional support, leveraging their existing social networks [51, 61]. Research found that domestic students benefited from community support groups and peer counselling programs provided by universities [55]. International students, on the other hand, faced unique challenges in accessing mental health support due to lack of awareness of resources on top of cultural differences and language barriers. As most international students are unaware of the need for and importance of mental health support, they chose not to report it and seek help. While there are studies that recommend tailored mental health services for international students, much of this research is found in literature that focuses exclusively on this population [44, 46, 47]. There remains a need for more comparative studies that explore how mental health services can address the distinct needs of both international and domestic students.

## Discussion

This scoping review, grounded in the WHO Commission on the Social Determinants of Health Framework, provides an in-depth analysis of how structural (e.g., student status—international vs. domestic) and intermediary determinants (e.g., food insecurity, housing issues, psychological factors) shape the experiences and wellbeing of international university students in comparison to domestic students.

The findings from this review revealed that university students, particularly international students, face elevated rates of food insecurity, social isolation, and psychological distress. Although housing issues are less discussed in the literature, they are emerging as a significant concern, particularly for international students. The interaction between these SDoH act as vulnerabilities, intensifying the impact on students’ wellbeing. These factors not only act independently but also interrelate, amplifying the negative effects on wellbeing. A systematic review in the United Kingdom (UK) similarly analysed vulnerabilities, buffers and triggers affecting the mental health of university and college students which found that strong social networks and early intervention improve mental health, while lack of engagement and poor mental health literacy increase vulnerability [63]. The WHO framework helps us understand how these determinants influence wellbeing and how social cohesion and social capital, the key aspects of wellbeing such as social support and inclusion, are critical in shaping wellbeing outcomes for students [37]. International students, facing greater cultural, financial, and social challenges, experience these issues more acutely than domestic students [64–67]. This review detailed the complex landscape and interplay of social determinants affecting international students, with more than 87% of the studies published in the last five years.

An important finding highlighted in this review is the role of mental health literacy and cultural differences in shaping the experiences of international students. While many international students face significant psychological distress, cultural norms and stigma often influence their willingness to seek help or discuss mental health concerns. A systematic review study conducted in Australia identified factors such as cross-cultural transitions, language barriers, social interaction, and mental health literacy [68]. Another cross-sectional study of domestic and international students found that international students had lower mental health literacy, help-seeking attitudes, and intentions to seek help for suicidal ideation, highlighting the need for targeted educational interventions [69]. These factors not only exacerbate feelings of isolation but also limit their access to mental health services, further impacting their overall wellbeing. The same cross-sectional study found that international students often resorted to coping strategies like avoiding or suppressing their difficulties instead of seeking appropriate support services [69].

In this review, social isolation has appeared as a major factor affecting the wellbeing of international students, and its significance has been increasingly discussed since the COVID-19 pandemic. A study conducted during the first peak of COVID-19 reported higher than average levels of anxiety and loneliness among university students, identifying lack of connection and social isolation as key risk factors for psychological distress in this population [70]. Another qualitative study conducted in the UK revealed similar findings showing that international students struggled to adapt to the new environment and experienced intensified feelings of loneliness [71]. Our results also indicate that the lack of social networks and support systems has not only been related to heightened levels of loneliness and disconnection but has also been closely linked with food insecurity. This aligns with another study indicating that psychological distress and food insecurity often co-exist among those with greater social isolation and weaker community belonging [72]. Experiences of food insecurity worsen due to limited resources and support networks, reducing shared resources and help-seeking behaviour, as shown in a narrative review on its impact on mental wellbeing [73]. This isolation also negatively affects psychological wellbeing, making it harder to manage food effectively and navigate logistical barriers to food assistance [73]. Similar findings were observed in another study exploring the impact of social isolation and loneliness on people’s health and wellbeing [74]. The interplay between social isolation and food insecurity creates a cycle of distress, where socially isolated students struggle to access food resources, face psychological and emotional challenges affecting the wellbeing [10, 75].

Housing issues are another critical issue in this review that significantly impacts the wellbeing of international students [76]. Our results showed that limited access to stable and affordable housing contributes to stress and anxiety, compounding other SDoH such as food insecurity and social isolation. A recent systematic review on the association between housing conditions and psychological distress among higher education students revealed a link between living arrangements and psychological distress, highlighting struggles with homesickness and substance abuse [77]. Another study conducted on international students in Japan found that poor housing conditions were statistically associated with a higher risk of developing depressive symptoms [78]. However, the connections between housing issues, food insecurity and social isolation yet remains underexplored in relation to international students. Additionally, limited research exists comparing the struggles around housing issues of international and domestic students and their impact on wellbeing. It is crucial to address this gap in the literature because unstable housing can limit students’ ability to maintain a healthy diet and connect with social support networks, further deteriorating their wellbeing and academic performance [79, 80].

For food insecurity, studies in this scoping review indicated that international students consistently face higher rates of low food security compared to domestic students. A cross-sectional study revealed that food security status impacts students’ psychosocial wellbeing [81]. Although several studies have explored the food insecurity status of international students, very few have made direct comparisons with domestic students, providing a deeper understanding of the extent and severity of the issue [79, 80]. Studies that find a higher prevalence of food insecurity among international students call for targeted interventions and support systems to address this critical issue [44, 45]. Overall, several studies in this review revealed that international students were more likely to experience food insecurity, social isolation, and housing issues than their domestic counterparts [44, 45]. This disparity may be attributed to limited financial resources, lack of access to health and social support systems, and the heightened vulnerability during the COVID-19 pandemic [26, 82].

In comparison to existing literature, our findings align with previous research on the adverse effects of food insecurity, social isolation, and housing issues on wellbeing. For instance, Goldrick-Rab et al. (2022) documented the increased food insecurity among American college students during the COVID-19 pandemic [82]. A review of basic needs among college students in the United States found that insecurities worsened during the COVID-19 pandemic, while Payne-Sturges et al. (2017) highlighted the significant link between food insecurity, wellbeing, and academic performance, corroborating our findings on international students’ vulnerability [83, 84].

This scoping review provides an understanding of the differential impact on international students compared to their domestic counterparts, revealing the unique and additional challenges they face. These challenges have been intensified by the COVID-19 pandemic, amplifying the financial, social, and psychological stressors for international students. The implications of these findings suggest that universities and policymakers should urgently implement comprehensive support systems that specifically address the unique challenges faced by international students. This includes providing more accessible wellbeing services, enhancing social support networks, and developing programs to ensure food security. For instance, studies have shown that targeted food assistance programs can significantly alleviate food insecurity and improve wellbeing outcomes [85]. Universities should also consider policies that facilitate better housing stability for international students, as housing issues has been demonstrated to be correlated with stress and psychological distress and affecting wellbeing [86]. Furthermore, enhancing social integration and support networks is crucial for mitigating the effects of social isolation. Policies aimed at fostering a supportive and inclusive environment, such as peer mentorship programs and culturally sensitive counselling services, can significantly improve the wellbeing and academic success of international students [87, 88]. By addressing these key SDoH, institutions can create a more inclusive and supportive educational environment that promotes the overall wellbeing, mitigate disproportionate impact on international students’ wellbeing and achieve academic success of all students.

### Limitations

This scoping review has some limitations. Firstly, the inclusion of studies with diverse methodologies and measures introduces variability, complicating direct comparisons and synthesis of results. Another limitation is the potential for publication bias. This review primarily includes peer-reviewed studies, which may overlook relevant data from unpublished studies or grey literature. Furthermore, the review is limited by its inclusion of only English-language studies, potentially excluding important findings from non-English publications. Finally, this review excluded studies focusing on higher education institutions in low-income and lower-middle-income countries (LICs and LMICs) due to differences in higher education systems, policies, and support structures in these regions. This decision ensured consistency in the study context and allowed for a more focused analysis. However, this exclusion may limit the generalisability of findings to these contexts. These limitations should be considered when interpreting the results of this scoping review, as they may impact the generalisability and completeness.

## Conclusion

This scoping review revealed significant challenges faced by international students in higher education, particularly about food insecurity, social isolation, housing issues and psychological distress compared to domestic students. These key SDoH are intricately linked to one another and collectively contribute to the vulnerability of these populations and affect their experiences and wellbeing. To address these issues, researchers emphasise the need for further longitudinal research to understand the long-term effects of food insecurity, social isolation, housing issues, and psychological distress on students. Additionally, future research should prioritise qualitative studies to explore the complex interplay between social determinants of health and in-depth investigations into housing issues to understand their impact on students’ wellbeing better. Research should also explore the coping mechanisms and resilience strategies of both international and domestic students to provide a more strengths-based perspective and inform targeted interventions.

There is a need for targeted interventions to enhance food security, improve housing stability, and provide financial and academic support, especially for international students who face unique challenges. Universities and policymakers must implement comprehensive support mechanisms, including accessible wellbeing services, enhanced social support networks, and programs to ensure food security and housing stability. Institutions are encouraged to develop culturally sensitive wellbeing services and peer support programs to address diverse student populations’ specific needs.

## Supplementary Information

Additional file 1: Supplementary Table 1. Measurement Instruments of Key Social Determinants of Health (SDoH) Psychological Distress, Psychological Wellbeing, Food Insecurity, Social Isolation and Social Support, Housing Issues, Others

Additional file 2: Supplementary Table 2. Summary of Key Findings

Supplementary Material 3. Prisma checklist

## Data Availability

The data will be made available upon request, in line with institutional guidelines and ethical considerations. To request access to the raw data analysed in this study, please contact the senior author Seema Mihrshahi at seema.mihrshahi@mq.edu.au.

## Abbreviations

GAD-7: Generalized Anxiety Scale
K10: Kessler Psychological Distress Scale
MeSH: Medical subject heading
MSPSS: Multidimensional Scale of Perceived Social Support
PD: Psychological distress
PHQ-9: Patient Health Questionnaire-9
PRISMA-ScR: Preferred Reporting Items for Systematic Reviews and Meta-Analyses extension for Scoping Reviews
PSS-4: Perceived Stress Scale
PTSD: Post-traumatic stress disorder
SDoH: Social Determinants of Health
USDA: United States Department of Agriculture
WHO: World Health Organisation
WHOQOL-BREF: World Health Organization Quality of Life Short Form
