# Supplementary material for: Unequal burdens: a scoping review of key social determinants of health affecting wellbeing of international vs. domestic students
Source: BMC Public Health. 2026 Jan 16;26:563. doi: 10.1186/s12889-025-26152-z (PMC12895619; doi:10.1186/s12889-025-26152-z)
Supplement: Supplementary file 1 — Additional file 1: Supplementary Table 1. Measurement Instruments of Key Social Determinants of Health (SDoH) Psychological Distress, Psychological Wellbeing, Food Insecurity, Social Isolation and Social Support, Housing Issues, Others [file 12889_2025_26152_MOESM1_ESM.docx]

**Additional file 1**

**Supplementary Table 1: Measurement Instruments of Key Social Determinants of Health (SDoH)**

**Psychological Distress, Psychological Wellbeing, Food Insecurity, Social Isolation and Social Support, Housing Issues, Others**

| **Reference Articles** | **Operationalization** | **Measurement Scales** |
| --- | --- | --- |
| **Psychological Distress: Depression** | | |
| Prado et. al., 2024; Amanvermez et. al., 2023; King et. al., 2023 | To assess depressive symptoms over the last 14 days | **Patient Health Questionnaire-9 (PHQ-9)**: Anxiety symptoms and depressive symptoms were measured with Nine items on a 4-point Likert scale ranging from 0 = “not at all” to 3 = “nearly every day.” The total sum score ranges from 0 to 27, and scores of 10 or more indicate clinically relevant symptoms and with higher scores meaning higher levels of depression. An example item from the PHQ-9 is “Feeling down, depressed, or hopeless”. Depressive symptom severity is classified as; minimal (0-4), mild (5-9), moderate (10-14), moderately severe (15-19), and severe (20-27). Scores ≥10 indicate positive screens for clinically significant symptoms. |
| Russell et. al., 2023 | To assess depressive symptoms over the last 14 days | **Patient Health Questionnaire 2 (PHQ2)**: 2-item PHQ-2 comprises the items ‘Over the last two weeks how often have you had little interest or pleasure in doing things’ and ‘Over the last two weeks how often have you felt down, depressed, or hopeless. The response options were ‘not at all, ‘several days’ ‘more than half the days’ and ‘nearly every day’. The two items were summed to obtain a scale score, with a recommended cut-off of three or more indicating probable major depression. |
| Kivelä et. al., 2022; Rekenyi et. al., 2023 | To screen for depression and to measure behavioral manifestations and severity of depression | **Beck Depression Inventory (BDI-I)**: A 21-item measure of current depressive symptoms. Total scores range from 0 to 63, with scores 0–9 reflecting none-to-minimal depressive symptoms, 10–18 mild-to-moderate, 19–29 moderate-to-severe, and 30–63 severe depressive symptoms. |
| Rekenyi et. al., 2023 | To identify these atypical or externalising symptoms in depressed males by adding distress symptoms to standard symptoms of depression | **Gotland Male Depression Scale (GMDS)**: 13 items, and it assesses not only the so-called “traditional” depressive symptoms but male depressive symptoms as well (lower stress threshold, aggression, substance abuse, over-involvement in work or sports). The respondents used a four-point Likert scale to answer the questions. The recommended point limits for the GMDS are respectively 0–13, 14–26, and 27–39. |
| Bennett et. al., 2022 | To assess a respondent's level of emotional distress caused by depressed mood in the past 7 days | **Patient-Reported Outcomes Measurement Information System (PROMIS) Depression and Anxiety Eight-Item (8a) Scales**: The item banks assess self-reported negative mood (sadness, guilt), views of self (self- criticism, worthlessness), and social cognition (loneliness, interpersonal alienation), as well as decreased positive affect and engagement (loss of interest, meaning, and purpose). Each question is rated on a five-point scale from 1=Never to 5=Always. |
| Dingle et. al., 2024 | To assess experiences of depression, anxiety and somatic distress in last 30 days | **Four items (each) on the PsyCheck for Depression, Anxiety and Somatic Distress**: Respondents are asked whether they had experienced each symptom over the past 30days, scoring yes=1 or no=0. Item scores were summed to pro- duce a score in the range of 0–4. |
| Dingle et. al., 2022 | To assess mental health in the last 30 days | **20-item PsyCheck Screening measure:** To assess mental health including somatic symptoms, in which research indicates to be relevant to many international student presentations. Participants responded ‘Yes’ or ‘No’ to questions asking whether they have experienced various physical and psychological symptoms in the last 30 days. |
| **Psychological Distress: Stress** | | |
| Prado et. al., 2024; King et. al., 2023 | To assess the perception of stress | **Perceived Stress Scale (PSS-4)**: A 4-item Perceived Stress Scale used. Items were answered on a 5-point Likert scale from 0 = “never” to 4 = “very often,” with a total sum score ranging from 0 to 16. Higher scores indicate more perceived stress. |
| Bennett et. al., 2022 | To assess the perception of stress in the last 30 days | **Perceived Stress Scale (PSS-10)**: The 10-item PSS measures global perceived stress experienced across the past 30 days on a 5-point scale (0 – never, 1 = almost never, 2 = once in a while, 3 = often, 4 = very often). |
| Amanvermez et. al., 2023 | To assess patterns, predictors, and consequences of midlife development in the areas of physical health, psychological well-being, and social responsibility | **Midlife Development in the U. S. (MIDUS)**: MIDUS is a self-report scale on a 5-point assessment with scores ranging between 0 and 4, meaning none, mild, moderate, severe, and very severe stress, respectively. This scale measures the current perceived stress during the data collection at several domains: financial situation, health, love life, relationship with family, relationship with people at work or school, the health of loved ones, other problems experienced by loved ones, and life in general. |
| Dana et. al., 2023; Larcombe, W. et. al., 2023; Kamardeen, I. and Sunindijo, R.Y., 2018 | To assess the severity of core groups of depression, anxiety, and stress and is not to diagnose psychological-related distress | **Depression, Anxiety, and Stress Scale (DASS-21)**:  Items measured the prevalence of symptoms of depression, anxiety, or stress with a four-point Likert scale (ranging between 0 = Never and 3 = Almost always). In brief, each area was classified into five levels, indicating normal (0), mild (1), moderate (2), severe (3), and extremely severe (4) problems. The scores for depression, anxiety, and stress each ranged from 0 to 42 (multiplying summed scores by 2): For depression, a total score of 0–9 was classified as normal, 10–13 as mild, 14–20 as moderate, 21–27 as severe, and 28–42 as extremely severe. For anxiety, a raw score of 0–7 was classified as normal, 8–9 as mild, 10–14 as moderate, 15–19 as severe, and 20–42 as extremely severe. For stress, a score of 0–14 was classified as normal, 15–18 as mild, 19–25 as moderate, 26–33 as severe, and 35–42 as extremely severe |
| Mihrshahi et. al., 2022; Skromanis et. al., 2018 | To measure of psychological distress and level of distress | **Psychological distress: Kessler psychological distress scale (K10)**: Participants were grouped into the following four levels of psychological distress: Low (scores of 10–15, indicating little or no psychological distress); Moderate (scores of 16–21); High (scores of 22–29); Very high (scores of 30–50). |
| **Psychological Distress: Anxiety** | | |
| Kivelä et. al., 2022 | To assess the intensity of physical and cognitive anxiety symptoms during the past week | **Beck Anxiety Index (BAI)**: A 21-item scale. Total scores range from 0 to 63, with scores 0–7 reflecting minimal anxiety, 8–15 mild, 16–25 moderate, and 26–63 severe anxiety |
| Russell et. al., 2023 | To measure or assess the severity of generalised anxiety disorder | **Generalized Anxiety Scale 2 (GAD-2)**: 2-item GAD-2 comprises the items ‘Over the last 2 weeks, how often have you been bothered by feeling nervous, anxious or on edge’ and ‘Over the last 2 weeks, how often have you not being able to stop or control worrying’. Response options were the same as those in the PHQ-2. Scale scores was the sum of these items, with a recommended cut-off of three or more indicating probable generalized anxiety disorder |
| Amanvermez et. al., 2023; King et. al., 2023 | To measure or assess the severity of generalised anxiety disorder | **Generalized Anxiety Scale (GAD-7)**: This scale consists of seven items, with each item ranging from 0 to 3. A sample item from the GAD-7 is “becoming easily annoyed or irritable”. Higher scores suggest high levels of anxiety. In the original work, the severity of the anxiety symptoms was categorized as minimal (0-4), mild (5-9), moderate (10-14), and severe (15-21). Scores ≥10 indicate positive screens for clinically significant symptoms. |
| Kivelä et. al., 2022 | To assess suicidal ideation in current (ie, past week) | **Beck Scale for Suicide Ideation (BSSI)**: A 21-item scale. Total scores range from 0 to 42, with higher scores reflecting more severe suicidal ideation. |
| Prado et. al., 2024 | To screen, diagnose, monitor and measure the severity of depression | **Item 9 of Patient Health Questionnaire-9 (PHQ-9)**: The presence of suicidal thoughts was assessed through item 9 of the PHQ-9 (“thoughts that you would be better off dead, or of hurting your- self”) with a score of 1 on a scale from “0 = not at all,” 1 = “several days,” 2 = “more than half the days,” to 3 = “nearly every day.” |
| Kivelä et. al., 2022 | To assess post-traumatic stress disorder (PTSD) symptoms | **PTSD Checklist for DSM-V (PCL-5):** This is a 20-item measure of DSM-V PTSD symptoms, with total scores ranging from 0 to 80. Scores above 33 are indicative of probable PTSD. |
| Kivelä et. al., 2022 | To assess academic stress | **Law Student Perceived Stress Scale (LSPSS):** LSPSS consists of 16 items assessing the impact of academic demands, career pressures, and study/life imbalance. Total scores range from 16 to 80, with higher scores reflecting more severe academic stress. |
| **Psychological Wellbeing** | | |
| Bennett et. al., 2022; Shi Y and Allman-Farinelli M., 2023 | To assess psychological wellbeing and mental health | **World Health Organization (WHO) - 5 Psychological Well-being Index**: This is a short, self-administered measure of well-being over the last two weeks (15, 16). It consists of five positively worded items that are rated on 6-point Likert scale, ranging from 0 (at no the time) to 5 (all of the time). |
| Dingle et. al., 2024; Dingle et. al., 2022 | To enable the monitoring of mental wellbeing in the general population and the evaluation of projects, programmes and policies which aim to improve mental wellbeing | **The Short Warwick–Edinburgh Mental Well- being Scale**: This comprises seven positively worded items such as I have been feeling useful. Participants rated the frequency they have had those symptoms over the last 2 weeks (1=none of the time to 5=all of the time). The scores are summed to give a total score in the range of 7–35, with higher scores representing better mental health and wellbeing. |
| Dingle et. al., 2024; Rosa D. et. al., 2023 | To assess emotional wellbeing via a series of questions and includes a physical examination | **Mental health screening**: 20-item measure that is quick to administer and assesses symptoms of depression and anxiety and somatic symptoms such as headaches or upset stomach. Endorsed items are summed to provide a score out of 20. Scores above 5 indicate a positive screen, indicating a recommendation for further assessment and management of a mental health issue |
| Yeung et. al., 2022 | To assess self-reported mental health diagnosis | **Self-report of having been diagnosed or treated by a professional within the past 12months for 15MH diagnoses**: Anorexia, anxiety, attention-deficit/hyperactivity disorder (ADHD), bipolar disorder, bulimia, depression, insomnia, other sleep disorder, obsessive-compulsive disorder (OCD), panic attacks, phobia, schizophrenia, substance abuse or addiction (alcohol or other drugs), other addiction (e.g., gam- bling, Internet, sexual), and other MH condition. For each listed condition, participants could endorse “no”; “yes, diagnosed but not treated”; “yes, treated with medication”; “yes, treated with psychotherapy”; “yes, treated with medication and psychotherapy”; or “yes, other treatment.” |
| King et. al., 2023 | To measure young adults' college-specific wellbeing | **College Student Subjective Well-Being Questionnaire subscale**: Low connectedness was defined as being in the bottom quartile of the sample distribution (subscale score ≤13). Cumulative Grade Point Average (GPA) was abstracted from the university database, with a GPA <2.7 over the first year considered as lower academic performance. |
| Skromanis et. al., 2018; Rosa D. et. al., 2023 | To assess subjective health and well-being | **Satisfaction with Life Scale:** A five-item measure designed to provide a brief assessment of the individual’s perceived overall satisfaction with their life. Item (and domain) scores ranged from one to five, with higher scores indicating greater satisfaction. |
| Smith et. al., 2022 | To assess psychological needs | **Psychological needs were measured by four items:** Sleep was measured via two-items, Healthy food intake was measured by a single-item; Safe living environment was also measured by a single item through 1–7 Likert-type scale from “strongly disagree” to “strongly agree. |
| Larcombe, W. et. al., 2023 | To assess psychological wellbeing | **18-item Ryff’s scales of Psychological Well-Being**: This measures six dimensions of positive psychological functioning: Autonomy; Environmental mastery; Personal growth; Positive relations with others; Purpose in life; and Self-acceptance. Respondents were asked to rate their level of agreement with items on a 7-point Likert-type scale ranging from ‘Strongly agree’ to ‘Strongly disagree’. |
| **Food Insecurity** | | |
| Bennett et. al., 2022; Mihrshahi et. al., 2022 | To measures the extent and experiences of food insecurity and hunger | **USDA Household Food Security Survey Module: Six-Item Adult Short Form:** The number of affirmative responses to questions to provide a raw score to categorising adult food security - high food security (score of 0), marginal food security (score of 1), low food security (score of 2–4) and very low food security (score of 5–6). |
| Hanbazaza et. al., 2017 | To measures the extent and experiences of food insecurity and hunger | **10-item Adult Food Security Survey Module:** Responses of “yes,” “often,” “sometimes,” “almost every month,” and “some months but not every month” are coded as affirmative. The sum of affirmative responses to the 10 questions in the Adult Food Security Scale is the household’s raw score on the scale. Food security status is assigned Raw score zero—High food security among adults, Raw score 1-2—Marginal food security among adults, Raw score 3-5—Low food security among adults, Raw score 6-10—Very low food security among adults. For some reporting purposes, the food security status of the first two categories in combination is described as food secure and the latter two as food insecure. |
| Dana et. al., 2023; Shi Y and Allman-Farinelli M., 2023 | To measures the extent and experiences of food insecurity and hunger | **United States Department of Agriculture Household Food Security Survey Module (USDA HFSSM) 18 item:** HF- SSM’s 18 questions capture and distinguish the various levels of severity of food insecurity and take into account the impact on dependent children**.** Food security status was categorized based on raw scores of the items asked, and if there are children in the household, eight child-related questions were included in the raw score. For students with one or more children, out of the maximum raw score of 18, a raw score of zero was classified as high food security, 1–2 as marginal food security, 3–7 as low food security, and 8–18 as very low food security [26,44]. For students without children, out of the maximum raw score of 10, a raw score of zero was classified as high food security, 1–2 as marginal food security, 3–5 as low food security, and 6–10 as very low food security [26,44]. Students with high or marginal food security were grouped as ‘food secure’ while those with low or very low food security were grouped as ‘food insecure’ |
| **Social Isolation and Social Support** | | |
| Prado et. al., 2024; Dingle et. al., 2022 | To assess experienced loneliness | **UCLA 3-Item Loneliness Scale**: 4-point Likert, from 0 = “never” to 3 = “often” [21].The total sum score ranges from 0 to 9 and higher scores indicate more loneliness experienced |
| Kivelä et. al., 2022 | To assess experienced loneliness | **De Jong-Gierveld Loneliness Scale (DJGLS)**: DJGLS consists of 11 items assessing subjective loneliness rated on a 5-point scale. Total scores range from 0 to 11, with scores 0–2 reflecting no loneliness, 3–8 moderate loneliness, and 9–11 severe loneliness. |
| Bennett et. al., 2022; Marczuk, A., & Lörz, M., 2023 | To assesses perceptions of being avoided, excluded, detached, disconnected from, or unknown by, others | **PROMIS Social Isolation Short Form:** Each question has five response options ranging in value from one to five. To find the total raw score for a short form with all questions answered, sum the values of the response to each question. For example, for the 4a short form, the lowest possible raw summed score is 4; the highest possible raw summed score is 20. This method of scoring uses responses to each item for each participant. This is referred to as “response pattern scoring.” Because response pattern scoring is more accurate than the use of raw score/scale score look up tables included in the manual, it is preferred. |
| Prado et. al., 2024 | To assess social support | **Five-item ENRICHED Social Support Inventory (ESSI)**: Five items answered on a 5-point Likert scale from 1 = “none of the time” to 5 = “all of the time,” with a total sum score ranging from 5 to 25. Higher scores indicate higher levels of social support. |
| Russell et. al., 2023 | To measure social support in patients with chronic disease at the primary health care | **MOS Social Support Survey (MOS-SSS-6)**: MOS-SSS-6 consists of six items gauging the presence of social support. Example items include ‘Someone to share private worries and fears’ and ‘Help if you are con- fined to bed’. A five-point scale is used to indicate how often these social supports are available (ranging from ‘none of the time’ to ‘all of the time’). The sum of the items ranged between 6 and 30, and to aid interpretation was dichotomized as “less than 18” vs “18 or more”, with higher total score indicating greater perceived support. The MOS-SSS-6 has been found to have a Cronbach’s alpha of 0.70 and reliability of 0.89. |
| Smith et. al., 2022; LaMontagne, A.D., et. al., 2023 | To assess key aspects of social relationships across the developmental spectrum | **Social relationships Scale:** This were measured via five separate items adapted from surveys derived from National Surveys such as the Canadian University Survey Consortium (CUSC) and National Survey of Student Engagement (NSSE) through a 1– 7 Likert-type scale from “strongly disagree” to “strongly agree. |
| Bennett et. al., 2022 | To assess perceived feelings of being cared for  and valued as a person; having confidant relationships. | **PROMIS Emotional Support Short Form**:  Each question usually has five response options ranging in value from one to five. To find the total raw score for a short form with all questions answered, sum the values of the response to each question. For example, for the v2.0 adult 4-item form, the lowest possible raw score is 4; the highest possible raw score is 16. This method of scoring uses responses to each item for each participant. This is refer to as “response pattern scoring.” Because response pattern scoring is more accurate than the use of raw score/scale score look up tables included in the manual, it is preferred. |
| Rekenyi et. al., 2023; Skromanis et. al., 2018 | To measure the extent to which an individual perceives social support from three sources: Significant Others, Family and Friends | **Multidimensional Scale of Perceived Social Support (MSPSS)**: The 12 items are rated on a seven-point Likert scale in the English version, while the 10-item Hungarian version uses a five-point Likert scale. Three subscales were identified, each addressing a different source of support: family, friends, and significant others |
| **Housing Issue** | | |
| Skromanis et. al., 2018 | To assess satisfaction with living conditions | **Environmental Health subscale of the World Health Organization Quality of Life Short Form (WHOQOL-BREF)**: 8 item measure designed to capture perceived satisfaction with living conditions among individuals from diverse cultural backgrounds. Item (and scale) scores ranged from one to five with higher scores indicating greater perceived satisfaction with one’s environment. |
