# Supplementary material for: Unequal burdens: a scoping review of key social determinants of health affecting wellbeing of international vs. domestic students
Source: BMC Public Health. 2026 Jan 16;26:563. doi: 10.1186/s12889-025-26152-z (PMC12895619; doi:10.1186/s12889-025-26152-z)
Supplement: Supplementary file 2 — Additional file 2: Supplementary Table 2. Summary of Key Findings [file 12889_2025_26152_MOESM2_ESM.docx]

**Additional file 2**

**Supplementary Table 2: Summary of Key Findings**

| **Author, Publication year** | **Food Insecurity** | **Social Isolation** | **Housing Issues** | **Psychological Distress and Wellbeing** | **Others (Academic Pressure, Pandemic Stressors, Financial Stressors)** |
| --- | --- | --- | --- | --- | --- |
| **Quantitative Studies** | | | | | |
| Prado et. al., 2024 | - | Increased level of loneliness over time; Statistically significant interaction effect for loneliness; Statistically significant effect of student status (domestic and international students) on dependent;  **Domestic Students:** Highest level of loneliness;  **International Students:** Lower level of loneliness despite lower social support than domestic students; Higher self-efficacy and resilience than domestic | - | Statistically significant effect of student status (domestic and international students) on dependent variables: depressive symptoms;  **Domestic Students:** Highest levels of depressive symptoms in 2021, but suicidal thoughts most prevalent in 2022;  **International Students:** Poorer mental health along with more depressive symptoms and perceived stress than domestic students; High levels of depressive symptoms and suicidal thoughts than domestic students; Consumed less alcohol than domestic students | **Domestic Students:** Worsened mental health and social and emotional aspects since pandemic; Increased prevalence of suicidal thoughts; Negative thoughts triggered by deaths from COVID-19;  **International Students:** Less affected by pandemic as had poorer mental health |
| Kivelä et. al., 2022 |  | **Domestic Students:** 10% of Dutch students indicated loneliness;  **International Students:** 25% of international students indicated severe loneliness; Faced unique stressors moving abroad alone at a relatively young age; Experienced social isolation and disconnect coping with new culture |  | **Domestic Students:** 9% had moderate-to-severe depressive symptoms; 12% had probable PTSD; 9% had severe anxiety;  **International Students:** 20% had moderate-to-severe depressive symptoms; 30% had probable PTSD; 25% had severe anxiety; Heightened mental health complaints; More depressive symptoms, suicidal ideation, anxiety, PTSD, academic stress and loneliness in comparison to Dutch students | increased fear of COVID-19 was associated with more depressive symptoms;  **International Students:** More fear of COVID-19 than domestic students |
| Russell et. al., 2023 | **Domestic Students:** Comparatively less food insecurity faced than international students; **International Students:** Fivefold increased odds of being unable to afford food during the pandemic than local students; 16% unable to afford food in the previous months; 16% ran out of food and could not afford to buy more | **Domestic Students:** Lack of social connection with the university, friends, and family at slightly higher rates than international students;  **International Students:** Worsened connection with peers and university during the pandemic; 70% Experienced low social support; Lower support than local students | **Domestic Students:** Majority lived with family or partner during pandemic;  **International Students:** More difficulty finding suitable accommodation; Lived alone without social support | **Domestic Students:** Worsened access to general health and mental health services;  **International Students:** Increased major depression; 1.5 times greater risk of further increase in depression and anxiety during pandemic than local students | **International Students:** Faced race-based discrimination |
| Amanvermez et. al., 2023 |  | **International Students:** Higher personal and social difficulties due to transition to new cultural and geographical environment; Lack of social support, cultural knowledge and language barrier than domestic; experience increased daily hassles, difficulties in socialization, or feelings of alienation and loneliness |  | **Domestic Students:** Find same situation less stressful as have more resources;  **International Students:** Significantly lower perceived stress related to health; Increase stress level due to experience of social changes, cultural and educational disparities; Higher emotional/ psychological complaints; Higher stress due to financial pressure; | **Domestic Students:** High financial burden but have more part-job opportunities available to support;  **International Students:** Higher levels of stress pertaining to the COVID-19 pandemic than domestic students in the Netherlands; Higher financial concerns associated with financial stress; Limitation in work permit and fewer job opportunities with high tuition fee |
| Bennett et. al., 2022 | **Domestic Students:** Of the 18% experiencing food insecurity (low and very low food security) - approximately 18.8% - 17.5% were domestic students, of the 14% marginal food secure - 44.4% are domestic students  and of the 68% of students being high food security - 43.4% were domestic students;  **International Students:** 18% experienced food insecurity (low and very low food security) - approximately 48.3% - 52.4% were international students, of the 14% marginal food secure - 27% are international students  and of the 68% of students being high food security - 16.8% were international students; Unemployed, living alone and postgraduate int students have severe  food insecurity | **International Students:** Not eligible for government's social support schemes; | **International Students:** Need to adjust with living alone; Increased living cost | **International Students:** Increased risk of depression and stress with detoriating food security status; stress and anxiety is inversely associated with food insecurity; Associated high level of stigma with food bank/pantry, triggers further stress and affect mental wellbeing; Food security negatively correlated with wellbeing | **International Students:** Food insecurity  related to poorer academic performance and affects mental health status; Despite having adequate food and financial literacy, they struggle as expenses exceeded income; COVID-19 put on additional stress on students in terms of online classes, travel restrictions and financial burden |
| Dana et. al., 2023 | 48% (n = 101) experienced food insecurity;  **Domestic & International Students:**  9 times more food insecure than domestic students; Vulnerability of students with children; 83% (43 out of 52) of severe food insecure students are international; 90% (19 out of 21) of international students with children had low and very low food security; 20% of the domestic students with children were low and very low food secure |  |  | **Domestic and International Students:** Food insecurity associated with mental health problems; For each unit increase in depression level, the likelihood of experiencing food insecurity increased; Higher prevalence of food insecurity associated with higher levels of psychological distress; One unit higher level of depression being 1.62 times more likely to experience food insecurity; Higher levels of depression and anxiety reported among students who experienced very low food security than those who were food secure | **Domestic Students:** Governments welfare assistance;  **International Students:** COVID-19 disproportionately impacted international students; High levels of hospitality  and accommodation industry job losses (e.g., stay-at-home orders and business closures) impacted ability to work |
| Dingle et. al., 2024 |  | **Domestic Students:** Levels of loneliness and sense of belonging at university were more problematic for domestic than international students; Main causes of stress - group assignments, studying for  exams, maintaining a healthy lifestyle, friendship issues  and mental health issues;  **International students:**  increased sense of loneliness and social alienation due to transition to overseas from collectivist cultures | **Domestic Students:** Mostly living with family;  **International Students:** Mostly living away from home in a share house or some form of student accommodation. Exception, during COVID-19 outbreak (2020, 2021) some were living with family - studying from offshore | Increased level of anxiety among students due to rising costs of living, shortages of affordable rental accommodation and environmental disasters;  **International Students:** 46-67% increase in mental health problems of international students pre-COVID (2019) to the first wave of COVID-19 (2020); Depression which was higher in international students than domestic;  **Domestic Students:** Worse mental health than international students across cohorts; Higher distress and lower wellbeing than international students; Two thirds (66.4%) of domestic students screened positive for mental health problems on PsyCheck in 2019 | **Domestic and International Students:** COVID-19 pandemic associated with a spike in mental health problems in both domestic and international students; |
| Hanbazaza et. al., 2017 | **Domestic Students:** Prevalence of marginal (3.2%), moderate (51.6%), or severe (45.2%) household food insecurity was 100% for domestic students;  **International Students:** Prevalence of marginal (14.8%), moderate (37.0%), or severe (44.4%) household food insecurity was 96.2% for international students;  Both, coping strategies - purchasing university supplies; accessing food banks but are not the solution to student hunger | **Domestic and International Students:** Lack of food support from peer, friends or relatives; Less likely to request food from friends or relatives to alleviate their food insecurity due to geographical distance, lack of social support and variation in cultural norms | **Domestic Students:** Majority (67.7%) lived with others; students' primary income sources (government loans for domestic students) may have been inadequate to meet their educational (e.g., tuition, compulsory fees, textbooks) and living (e.g., food, rent, utilities) needs;  **International Students:** Majority (59.3%) lived with others; students' primary income sources (research assistantships for international students) may have been inadequate to meet their educational (e.g., tuition, compulsory fees, textbooks) and living (e.g., food, rent, utilities) needs | Food insecurity is associated with emotional distress and depression; **Domestic and International Students:** Better mental health of international students than domestic; Three possible underlying reasons - either healthier (undergo medical exam for immigration) or not recognizing the mental health problem or chosen not to report | Lack of financial support (only rely on research assistantship), no govt funds or loans available for int students |
| Yeung et. al., 2022 |  | **Domestic Students:** Stigma toward psychological help-seeking; Lack of access to culturally sensitive services;  **International Students:** Lack of family and campus belongingness than domestic students; Lack of social support network; Negatively associated with suicidal ideation; |  | **Domestic Students:** More aware of the severity of suicide;  **International students:** Higher risk of depressive symptoms and suicide attempts; Lower rates of diagnoses of other mental health issues including anxiety, comorbid anxiety and depression; Underutilize mental health services (illness beliefs, stigma, lack of awareness of the need for help, and lack of awareness of resources); Feel more overwhelmingly depressed than domestic students |  |
| King et. al., 2023 |  | **Domestic and International Students:** Both go through childhood adversity, lower self-esteem, lower social competence, higher perceived stress, substance misuse, lower social support, and a lower internal locus of control; Int Students have higher perceived stigma and lower mental health literacy than domestic |  | Both comparable rates of screening positive for depressive symptoms and lifetime suicidal thoughts at school entry;  **Domestic Student:** More likely to screen positive for anxiety and insomnia;  **International Students:** Lack of social support and isolation associated with an increased risk of screening positive for anxiety and depression; higher rates of  mental health symptoms among international students; Less likely to report a family history of mental illness, especially among females; Lower self-esteem and greater perceived stress; Stigma around mental health problems may be higher, fostering denial and lowering reporting of mental health problems | **International Students:** Lower school connectedness, have lower cumulative GPAs, and have failed one or more courses |
| Mihrshahi et. al., 2022 | **Domestic and International Students:** Higher level of food insecurity among international students (74.4%) compared to domestic students (22.7%); | **Domestic Students:** Returned to live with family homes during COVID-19,  **International Students:** Homesickness and loneliness due to travel restriction during COVID-19 |  | **International Students:** Significantly higher psychological distress scores than domestic students; K10 5.05 points higher than its average for domestic students; Food-insecure students had a significantly higher prevalence of high psychological distress than food-secure students | **Domestic Students:** Improved food security due to moving in with family during the  COVID-19 pandemic |
| Rekenyi et. al., 2023 |  | Female students receive more support than males; **Domestic Students:** More social support in all areas than their international counterparts, but social support seemed to level off between domestic and international students as a result of the increasing time spent under the threatening factors; **International Students:** Significantly lower values in terms of perceived social support; Lack of social support from family, friends, and also special persons in their lives |  | Higher level of depression among international female students than domestic female students; International students are prone to being exposed to more psychological stressors because of the separation from their home environment and their different cultural values, language, levels of academic preparation, and study habits; Lack of social support leads to isolation and loneliness which leads to more suffering from depressive symptoms among int students |  |
| Shi Y and Allman-Farinelli M., 2023 | **Domestic students:** 13.0% food insecure; Lower fruit intake was reported by food-insecure domestic students; undergraduate students had higher odds of being food insecure than postgrad, students in the fourth year or above of their degree were more likely to experience food insecurity,  **International Students:** 18.7% food insecure; Higher odds of being food insecure than domestic students; Twice likely to experience food insecurity than domestic students during the COVID-19 pandemic; More likely to use food assistance services than food-insecure domestic students |  | Moved to less-expensive premises during pandemic reported in both domestic and international students; **Domestic Students:** During pandemic, move back to their parental homes; Students living in accommodations other  than the parental home had an elevated risk of food insecurity; **International Students:** Struggles with living arrangements during pandemic which increased food insecurity; accommodation with meals included had higher odds of food insecurity than those living in accommodations without provided meals | Both domestic and international students with food insecurity was indicating poor well-being; Poor mental health and triggered stress and anxiety have been reported for both groups | Students who lost employment income because of the COVID-19 pandemic were more likely to be food insecure; None of the food-insecure international  students received any financial support from the government during the pandemic compared with 57% of food-insecure domestic  students. |
| Skromanis et. al., 2018 |  | **Domestic and International Students:** Lower levels of support from significant others and friends than domestic students; Social support networks left behind; Difficulty in establishing comparable relationships in a new country; Poorer global life satisfaction, poorer perceived social support, greater dissatisfaction with their environmental circumstances, higher levels of smoking and illicit drug use, and higher levels of problem gambling behaviors, than domestic students |  | No significant differences between groups in terms of psychological distress; **Domestic Students:** More likely than international students to rate their health as being fair or poor;  More than half of domestic students had sought help for a mental health or related problem whereas less than one in five international students had sought such help;  **International Students:** Mental health help seeking behavior - int students less likely to report than domes students; Lack of understanding among int students of severity of psychological distress; Poorer social support leads to poorer mental health; gendered dimension - females had poorer health |  |
| Smith et. al., 2022 | **Domestic Students:** More likely to report believing having access to healthy food;  **International students:** Less confidence in having access to healthy food compared to domestic students; Utilization of food bank - over 60% of students | **Domestic and International Students:** No significant differences between int and domes students in terms of perceived important outcomes of building relationships with others and ability to connect with family and friends; Recognise more the importance of strong social network in comparison to domestic students; Int students have higher intentions for campus engagement than domestic students | **Domestic Students:** More likely to having a safe place to live for the near future; **International Students:** Lack of confidence to find a secure place to live than domestic students. | Awareness around psychological needs critical international students; Vary substantially between domestic and international students; Barriers to psychological wellbeing are - not having - a safe place to live, access to healthy food, and adequate sleep; | International students: Limited resources and working opportunity |
| Dingle et. al., 2022 |  | **Domestic Students:** domestic students were lonelier than international students; Increase in loneliness in 2020 compared to 2019 and 2021; Decreased sense of university belonging due to restriction; **International Students:** Less loneliness than the domestic students - higher average age and confidence lead to stronger existing social networks |  | Lower university belonging and higher loneliness during the pandemic and this was detrimental to their mental health; Knowledge around access to health and well-being and by engaging in simple strategies such as socialising with others, eating well, sleeping well, physical activity, study skills, and emotion regulation psychological wellbeing can be improved; | Social restrictions (distancing, lockdown) impact social connectedness negatively |
| Larcombe, W. et. al., 2023 |  |  |  | Both domestic and international students go through anxiety and depression. There is no significant difference between level of anxiety faced by international and domestic students. However, there is a negative correlation between self compassion and depression-anxiety. People with more self compassion have a better psychological wellbeing. International students have proven to have more self compassion, thus have slightly low level of depression compared to domestic students. |  |
| Marczuk, A., & Lörz, M., 2023 |  | Social isolation during the COVID-19 pandemic contributed to delays in study progress, particularly due to difficulties in learning group exchanges, reduced contact with peers, and challenging communication with lecturers. This had a minimal impact on migration-specific differences, and overall, the effect of social isolation on study delays among German students was found to be minor. International students were more affected by this. |  |  | Financial difficulties, especially the worsening financial situation of international students' parents, were strongly linked to delays in study progress. International students reported higher rates of reduced financial support from their parents and more frequent job loss compared to German students, which significantly contributed to delays in academic progress. The pandemic exacerbated financial challenges, particularly for international students, whose parents, often living outside of Germany, experienced greater financial hardship. |
| LaMontagne, A.D., et. al., 2023 |  |  |  | Most respondents (83%) identified depression in the vignette, with international students being less likely than domestic students to do so (74% vs. 86%), even after adjusting for age and sex. 94% of all respondents identified at least one mental health problem, with no significant difference between international and domestic students. Nearly half (46%) of students reported experiencing a mental health problem in the past 12 months, with international students significantly less likely to report one compared to domestic students (26% vs. 52%). Among those reported a mental health issue, 70% sought help, with international students being less likely to seek help (62% vs. 71%) in bivariate analysis. |  |
| Kamardeen, I. and Sunindijo, R.Y., 2018 |  | International students face significant challenges related to adapting to a new environment, with stressors such as loneliness, homesickness, difficulties socializing with locals, and adjusting to new learning methods being prominent concerns. In contrast, local students experience fewer issues related to social isolation, as their primary stressors are related to family commitments. These adjustment-related stressors make international students more vulnerable to social isolation compared to their local counterparts. |  | Despite experiencing higher levels of stress, local students generally perform better academically than international students. Research shows that local students in Canada tend to outperform international students, although the gap in academic performance varies based on the ethnic background of international students. Anxiety, high self-expectations, and concerns about academic performance are prominent psychological stressors for both local and international students, further intensifying the pressures they face in their academic environments. | While both local and international students face academic stress, the nature of these stressors differs significantly. Local students report difficulties in balancing study and work demands as their top challenge, whereas international students are more concerned about finding a job after graduation. Both groups share six common top stressors, including anxiety about under-performing, high self-expectations, sleep deprivation due to workload, high academic demands, lack of social activities, and challenges in group assignments. |
| Rosa D. et. al., 2023 |  |  |  | This focus on associations between stress, emotional well-being, and physical activity among international and domestic students – Students reported generally moderate to high levels of stress and emotional well-being. Among students, 12% of international and 15% of domestic students met the recommended 150 minutes of moderate-to-vigorous physical activity (MVPA) per week, with no significant difference between the groups. Only 7.6% of international and 9.4% of domestic students met both MVPA, again showing no significant difference. International students reported significantly lower stress, emotional well-being, and fewer days of MVPA compared to domestic students, while there were no significant differences in the number of days of strength training. |  |
| **Qualitative Studies** | | | | | |
| Wright et. al., 2021 | **Domestic Students:** More than 60% of domestic students had low or very low food security scores; **International students:** 100% experienced cultural food insecurity in the last three years; Difficulty finding their cultural foods; Even when cultural foods were available, they often were not accessible because of cost; | **Domestic and International Students:** Both groups acknowledged importance of mealtimes to bond with friends and family.  Int students highlighted that the culture of family mealtimes Strengthened ties to home country, culture, and family. Lack of these leads to feelings of homesickness |  | Both group of students feelings of emotional discomfort when they experienced cultural food insecurity;  **International Students:** Mentally taxing trying to collect cultural foods; Grocery shopping and cultural food acquisition creates additional stressors as unfamiliar with navigating which is not the case for domestic students; Struggles with academic transition adds on to the stressors; Feelings of sadness, stress, and anxiety because they did not have many anchors back to their culture; Feelings of identity loss and disconnect |  |
| Aydın OT., 2020 |  | **Domestic and International Students:** Social relationships with students in the host country improve the experience of international students; Experienced language problems with local/Turkish peers; Cultural differences as identified by both groups of students in their communications with one another lead to loneliness, exclusion, and isolation; Introversion and shyness - lack of friendships |  | **Domestic and International students:** Feeling of anxiety and shyness to communicate with each other in a language which is not their first language; Poor language skills resulted in the anxiety and stress |  |
